# Immunogenicity of Bacillus Calmette-Guérin in pigs: potential as a translational model of non-specific effects of BCG

**DOI:** 10.3389/fimmu.2023.1219006

**Published:** 2023-07-13

**Authors:** Kristoffer Jarlov Jensen, Mette Sif Hansen, Kerstin Skovgaard, Erik Svensson, Lars Erik Larsen, Peter M. H. Heegaard, Christine Stabell Benn, Gregers Jungersen

**Affiliations:** ^1^ Bandim Health Project, University of Southern Denmark, Copenhagen, Denmark; ^2^ Department of Biotechnology and Biomedicine, Technical University of Denmark, Kgs. Lyngby, Denmark; ^3^ Copenhagen Phase IV Unit, Center for Clinical Research and Prevention and Department of Clinical Pharmacology, Copenhagen University Hospital – Bispebjerg and Frederiksberg, Frederiksberg, Denmark; ^4^ Institute for Veterinary and Animal Sciences, University of Copenhagen, Frederiksberg, Denmark; ^5^ Center for Diagnostics, Technical University of Denmark, Kgs. Lyngby, Denmark; ^6^ Department of Tuberculosis and Mycobacteria, Statens Serum Institut, Copenhagen, Denmark; ^7^ Department of Health Technology, Technical University of Denmark, Kgs. Lyngby, Denmark; ^8^ Danish Institute for Advanced Study, University of Southern Denmark, Odense, Denmark; ^9^ Department of Infectious Disease Immunology, Statens Serum Institut, Copenhagen, Denmark

**Keywords:** non-specific effects, heterologous immunity, pigs, BCG, *Actinobacillus pleuropneumoniae*, influenza virus

## Abstract

**Background:**

Clinical and immunological studies in humans show that the live attenuated *Bacillus Calmette-Guérin* (BCG) vaccine has beneficial non-specific effects, increasing resistance against diseases other than tuberculosis. The underlying mechanisms are currently being explored. The pig exhibits considerable physiological similarity to humans in anatomy and physiology, suggesting that similar responses to BCG could be expected. Studies of the non-specific effects of BCG in pigs are scarce. We investigated the feasibility of using pigs as a large animal model to investigate the non-specific immunological effects of BCG.

**Methods:**

In a series of experiments, we randomized newborn or young piglets from conventional farms to receiving BCG or placebo and investigated the persistence of live BCG bacteria in various tissues, the immunogenicity of BCG in *ex vivo* blood and *in vitro* stimulation assays, and the acute phase protein and clinical responses to heterologous infectious challenge with influenza A virus or *Actinobacillus pleuropneumoniae*.

**Results:**

The BCG vaccine was generally well tolerated. In contrast to humans, no skin reaction in the form of abscesses, ulcers, or scars was observed. Live BCG was recovered from draining lymph nodes in 2/13 animals 20 weeks after vaccination. Specific *in vitro* responses of IFN-γ to antigen-specific re-stimulation with mycobacterial antigen were increased but not TNF-responses to TLR2 or TLR4 agonists. A few genes were differentially expressed in blood after vaccination, including the antiviral genes *RIG-I* and *CSF1*, although the effect disappeared after correction for multiple testing. Clinical symptoms after heterologous bacterial or viral respiratory infections did not differ, nor did virus copies in nasopharyngeal samples after the challenge. However, the acute phase protein response was significantly reduced in BCG-vaccinated animals after influenza challenge but not after *A. pleuropneumoniae* challenge.

**Discussion:**

BCG was safe in pigs, inducing specific immunological responses, but our model did not corroborate the innate immunological responsiveness to BCG seen in humans. The dose of BCG or the bacterial and viral challenges may have been sub-optimal. Even so, the acute phase protein response to influenza infection was significantly reduced in BCG-vaccinated animals.

## Introduction

Human studies have demonstrated that the tuberculosis vaccine Bacillus Calmette-Guérin (BCG) derived from *Mycobacterium bovis* has non-specific effects, conferring partial protection against heterologous infections and related mortality (1). For example, three randomized trials in Guinea-Bissau found that early BCG vaccination reduced neonatal mortality by 38% (95% CI: 17-54%) in low-weight infants (2).

The immunological mechanisms behind the heterologous protective effects of BCG are starting to be understood, with modulation of innate immunity suggested to be involved. Data from mice and humans show that monocytes and NK cells exert stronger responses to innate stimulation after training with BCG *in vitro* as well as *in vivo* (3). Furthermore, BCG was shown to induce emergency granulopoiesis, possibly contributing to better immediate infection control (4). Despite the physiological similarity between pigs and humans (5, 6), few studies have used the pig as a large animal model to unravel the immunological effects of BCG.

If BCG vaccination has beneficial non-specific effects in pigs, these properties may reduce infectious disease mortality in modern pig farming. However, despite the high safety profile of BCG from billions of doses routinely administered to humans worldwide, administration of live BCG to pigs may cause concerns in the food industry regarding consumer safety and will interfere with surveillance and routine diagnostics for bovine tuberculosis. Nonetheless, BCG has been explored as an asset in the control of bovine tuberculosis in cattle both historically and more recently (7), and if more specific diagnostic tests to be used in parallel with BCG emerge, these restrictions may change in the future. In addition, as the non-specific effects of live vaccines is best established with BCG, the use of BCG in pigs may also be a relevant translational model to study non-specific effects of vaccines which may be taken into consideration in the design of new veterinary vaccines, a proposal already being pursued in human vaccinology (8).

This motivated us to investigate if live BCG 1) boosted innate immunity in pigs, 2) decreased susceptibility to heterologous infection, thereby exploring if pigs may serve as a useful animal model to explore the non-specific immunological effects of BCG, and finally, 3) could be detected in tissues of pigs at slaughter when immunized as newborns, thereby exploring the feasibility of using BCG or other products with similar potential beneficial properties in pig farming as an immunological booster.

## Materials and methods

**Figure 1:**
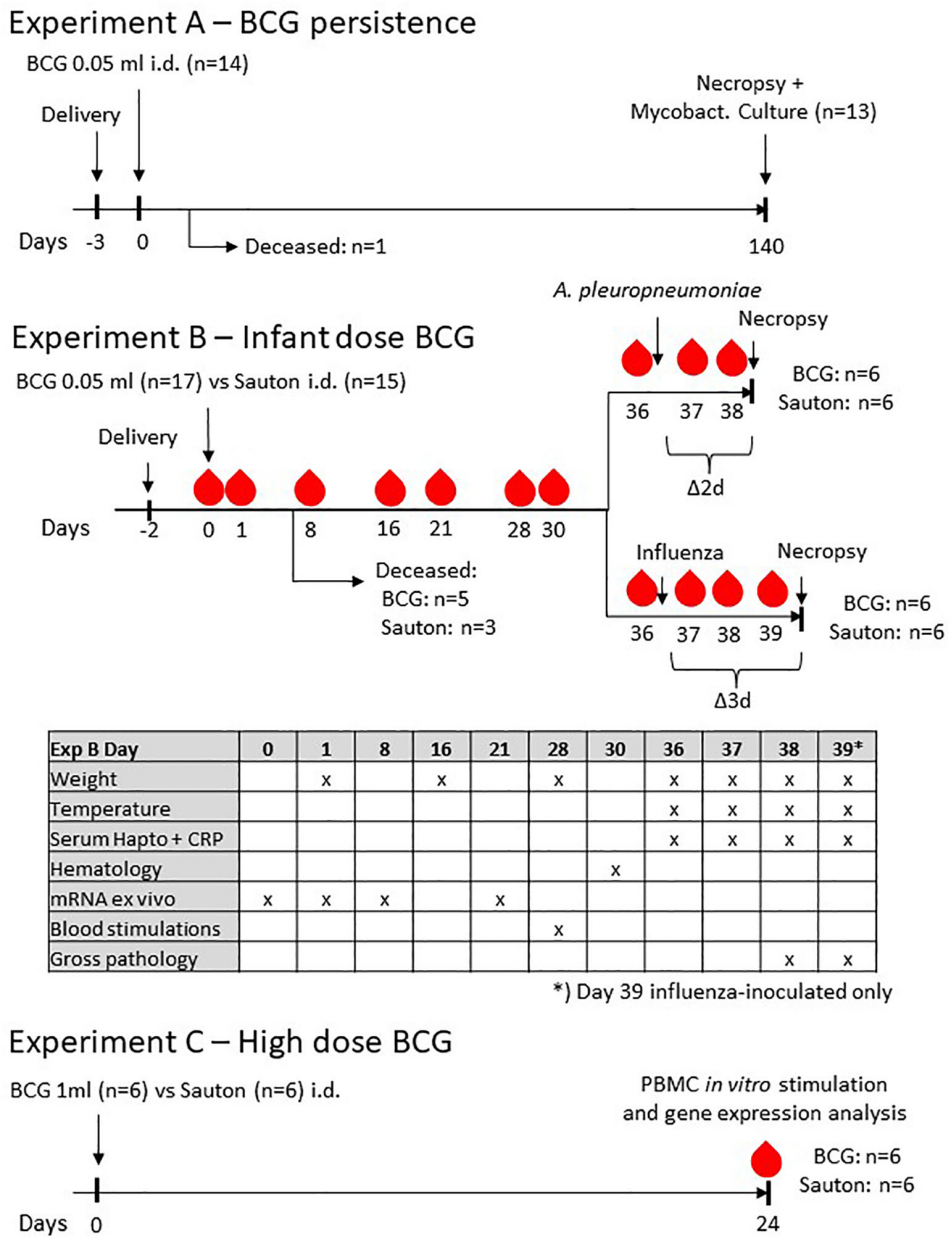
Experimental design of the three experiments.

The outline of the three animal experiments (Experiments A, B, and C) is found in **Figure 1**. All pigs in the present studies were Danish Landrace/Danish Yorkshire crossbreds and paternal line Duroc, born and raised in conventional commercial pig farms. The BCG used in all experiments was BCG Vaccine SSI (Danish strain 1331) as was the diluent Sauton (SSI Diagnostika, Hillerød, Denmark). For all experiments (A, B, and C), vaccination was on Day 0. All animals were sacrificed by captive bolt followed by bleeding.

### Experiment A

One pregnant sow was purchased from a commercial farm (Farm A) and delivered to the research facilities at Lindholm, Technical University of Denmark, Kalvehave, Denmark. All 14 piglets received 0.05 ml BCG intradermally (i.d.) in the skin of the right ear base three days after delivery. One piglet was found dead on day 9, presumably crushed under the sow. After 20 weeks (day 140), the remaining 13 pigs were euthanized by captive bolt, followed by bleeding. Tissue samples were collected in sterile falcon tubes and analyzed at the International Reference Laboratory of Mycobacteriology, Statens Serum Institut, Copenhagen, Denmark, for the presence of live BCG. Tissue samples were taken from the liver, lung, kidney, bone marrow from the left femur, muscle from the shoulder, superficial dorsal cervical lymph node, retropharyngeal lateral lymph node, and the skin at the vaccine injection site.

### Experiment B

Three pregnant sows were purchased from Farm A and delivered to the research facilities at Frederiksberg, National Veterinary Institute, Technical University of Denmark, Denmark. Two to three days after delivery, 32 piglets were randomized 1:1 within each of the three litters to receive i.d. 0.05 ml BCG (n=17) or 0.05 ml Sauton (BCG solvent used as a placebo, n=15) in the right shoulder, administered by a midwife with extensive experience in BCG vaccination of human newborns.

After BCG administration, weight was recorded on days 1, 16, 28, and 36-39, and rectal temperature on days 36-39.

On day 36, eight pigs had been lost (see the Results section for details), and the remaining pigs (n=24, BCG: 12; Sauton: 12) were allocated based on current weight into two groups, ensuring an equal distribution of BCG- and Sauton-treatments. One half (n=12, 6/6) was inoculated intranasally with *Actinobacillus pleuropneumoniae* 4226, serotype 2 (1 ml in either nostril as detailed below). The other half (n=12, 6/6) received 4 ml of10^6^ TCID_50_/ml swine influenza virus H1N2 (A/swine/Denmark/101394-1/2011(H1N2) using MAD Nasal™ intranasal mucosal atomization devices (Teleflex, Morrisville, NC, cat. no. MAD100) 2 ml in either nostril). Before inoculation, the animals were anaesthetized with i.m. injection of a mixture of 12.5 mg tiletamine, 12.5 mg zolazepam, 12.5 mg xylazine, and 12.5 mg ketamine per mL at 1 mL/15 kg. The *A. pleuropneumoniae* group was sacrificed on day 38 (two days after bacterial infection) and the influenza group on day 39 (three days after viral inoculation). Blood was collected in PAXGene tubes (2.5ml, Qiagen) on days 0, 1, 8, and 21 and stored at -20°C until further processing.

### Experiment C

For experiment C, 12 female pigs (Danish Landrace/Danish Yorkshire crossbreds and paternal Duroc) were delivered after weaning to the research facilities at Frederiksberg from a commercial farm (Farm B). At 5 weeks of age, the pigs were stratified by size (total weight range 5.5-11.5 kg, mean 8.9 kg) and allocated to two treatment groups, receiving either 1.5-2 vials of BCG resuspended in 0.8 ml Sauton (equaling 15-20 times a standard BCG dose, n=6) or 1.0 ml Sauton alone (n=6), applied by three adjacent injections i.d. in the right hind leg. 24 days after immunization, venous blood was collected into heparinized tubes for stimulation assays, and the pigs were sacrificed.

### Mycobacterial detection (Experiment A)

The tissue samples listed in the outline of Experiment A above were analyzed at the International Reference Laboratory of Mycobacteriology, Statens Serum Institut, Copenhagen, Denmark, for the presence of live *Mycobacterium bovis* BCG. Specimens were disintegrated with a scalpel and in a sample homogenizer (GentleMACS, Dissociator, Miltenyi Biotec, Bergisch Gladbach, Germany) and then pre-treated with MycoPrep (NALC-NaOH, Becton Dickinson, Sparks, MD). The pre-treated material was analyzed with microscopy, PCR specific for *Mycobacterium tuberculosis* complex (Cobas TaqMan MTB, Roche Diagnostics, Rotkreutz, Switzerland), and culture in MGIT (Becton Dickinson) and Löwenstein-Jensen (SSI Diagnostika) media. The growth of *M. bovis* BCG was verified with GenoType MTBC (Hain Lifescience, Nehren, Germany).

### *Actinobacillus pleuropneumoniae* (Experiment B)

A seed lot of *A. pleuropneumoniae* strain 4226, serotype 2, stored at -80°C was cultured overnight at 37°C on modified pleuropneumonia-like organism (PPLO)-agar plates using *E. coli* as a nurse strain, suspended in 0.9% NaCl, and adjusted to the desired concentration by turbidity of McFarland standard 0.5 diluted 1:1 in BHI+0.5% NAD. Animals were inoculated with 2 ml of *A. pleuropneumoniae* suspension, equaling 0.9x10^6^ CFU/animal. The concentration and purity of the inoculation suspensions were verified by seeding on PPLO and blood agar plates, respectively. After necropsy, for re-isolation of the inoculation strain from tissue samples (liver, spleen, and lung), a scalpel was inserted into the affected tissue and struck on PPLO agar and cultured at 37°C; the following day, emerging colonies were enumerated.

### Swine influenza A propagation and quantification (Experiment B)

Swine Influenza A H1N2 virus was propagated in Madin-Darby Canine Kidney Epithelial Cells (MDCK) and prepared for inoculation as previously described (9). Influenza A virus load was quantified from nasal swabs preserved in saline using RT-qPCR. Total RNA was extracted from 140 µl of the nasal swab sample by QIAamp Viral RNA Mini Kit (Qiagen) using the manufacturer’s procedure and stored at -80°C until analysis. The influenza A virus was detected by an in-house modified version of a real-time RT-qPCR assay detecting the matrix gene (10).

### Necropsy (Experiment B)

Necropsy was performed to characterize gross lesions in *A. pleuropneumoniae*-inoculated and influenza-inoculated pigs. The lungs were examined for *A. pleuropneumoniae*-like lesions defined as areas with hemorrhage, consolidation, necrosis of lung tissue, and fibrin exudation. Influenza lesions were defined as mucopurulent exudate in bronchi and bronchioles and lobular atelectasis of lung tissue.

### Acute phase proteins (Experiment B)

In Experiment B, from serum samples collected on days 16, 36, 37, 38, and 39 (day 39 only from influenza-challenged pigs), CRP and Haptoglobin were analyzed using in-house ELISA protocols (11, 12).

### Differential count and hematological chemistry (Experiment B)

In Experiment B, on day 30, a whole blood differential count was performed (ABC Vet, Horiba ABX, France) using EDTA-treated blood, yielding counts of leukocytes, red blood cells, and platelets, and concentrations of hemoglobin, mean corpuscular hemoglobin, and mean hematocrit percentages and mean corpuscular volume.

### *In vitro* stimulations (Experiments B and C)

In Experiment B, on day 28, undiluted whole blood was stimulated with LPS (10 ng/ml, from Escherichia coli O55:B5, Sigma-Aldrich), Pam3CSK4 (1 µg/ml, Invivogen), Staphylococcus enterotoxin B (1 µg/ml), phytohaemagglutinin (PHA) (2µg/ml), or PPD from *M. tuberculosis* (10 µg/ml, Statens Serum Institut) for 22h at 37°C, 5% CO_2_ humidified incubator.

In Experiment C, peripheral blood mononuclear cells (PBMC) were purified from the heparinized blood within 2 hours of venipuncture. Blood was diluted 1:1 in sterile PBS supplemented with 2% fetal calf serum (FCS) and carefully applied to a StemCell SepMate tube containing Lymphoprep density gradient medium (StemCell Technologies). After centrifugation, the PBMC-containing supernatant was retrieved and washed twice in PBS/2% FCS. To lyse red cells, the cell pellet was resuspended and incubated with red cell lysis buffer (78mM NH_4_Cl, 0.05 mM EDTA and buffered with 5mM KHCO_3_). The PBMCs were resuspended in RPMI-1640 Glutamax (Gibco) supplemented with 10% FBS and 1% penicillin/streptomycin, seeded out as 14x10^6^ cells/well in a 24-well Cellstar plate (Greiner Bio-One, Frickenhausen, Germany), and stimulated at 37°C, 5% CO_2_ with LPS (10 ng/ml final concentration, Sigma), Pam3CSK4 (1 µg/ml, Invivogen), Staphylococcus enterotoxin B (1 µg/ml, Sigma), Purified protein derivative (PPD) from *M. tuberculosis* (10 µg/ml, Statens Serum Institut), or medium alone. After 7h, the plates were centrifuged, supernatants removed, and the cell pellets harvested in RLT buffer (Qiagen) with 1% v/v beta-mercaptoethanol and stored at -80°C until RNA extraction.

### RNA extraction (Experiments B and C)

In Experiment B, blood was collected in PAXgene tubes, and extraction was performed according to the manufacturer’s instructions using the PAXgene Blood miRNA Kit (Qiagen). In Experiment C, RNA was extracted from PBMCs preserved in RTL buffer after *in vitro* stimulations using the QiaAmp RNA Blood mini protocol according to the manufacturer’s instructions (Qiagen). The quantity of the RNA was measured using a spectrophotometer (NanoDrop ND-1000, Thermo Scientific, United States). RNA integrity was measured on an Agilent 2100 Bioanalyzer (Agilent Technologies, Nærum, Denmark). RNA integrity number (RIN) values in Experiment B were in the range of 7 to 9, averaging 7.6; RIN values in Experiment C were in the range of 8.7 to 9.7, averaging 9.2.

Synthesis of cDNA was performed in duplicates from a total of 500ng RNA per reaction using QuantiTect Reverse Transcription Kit (Qiagen #205311) as previously described (13). Non-reverse transcriptase reactions were included as a control for possible genomic DNA contamination.

In Experiment B, the resulting cDNA concentration was measured on NanoDrop and diluted to 200 ng/μl in 20 μl in low EDTA TE-buffer before pre-amplification.

Fifteen cycles of pre-amplification were performed as previously described (13) using TaqMan PreAmp Master Mix (Applied Biosystems, PN 4391128), followed by Exonuclease I treatment (New England Biolabs, PN MO293L) of 30 minutes at 37°C and 15 minutes at 80°C.

qPCR was performed in 96.96 Dynamic Array Integrated Fluidic Circuits (Fluidigm, CA, USA) according to (14), combining 96 samples with 96 primer pairs (**Supplementary Table 1**). A non-template control and the non-reverse transcriptase controls from the cDNA synthesis were included on each array chip to monitor for non-specific DNA contamination, including of genomic origin.

Expression data (Cq values) and melting curves were acquired using the Fluidigm Real-Time PCR Analysis software 3.0.2 (Fluidigm) and exported to GenEx (MultiD, Sweden) for data pre-processing including censoring for non-specific amplification or low amplification, interplate correction, correction for PCR efficiency for each primer assay individually, normalizing using GeNorm and NormFinder programs according to the expression of ACTB, B2M, RPL13A, PPIA, TBP, and YWHAE in Experiment B and ACTB, B2M, GAPDH, RPL13A, TBP, and TGPI in Experiment C, and averaging of cDNA technical repeats. Data was scaled by setting the lowest expression of each gene of interest to 1 whereby the remaining samples were expressed as the ratio relative to this lowest sample. Experiment B data was also log2 transformed.

After censoring the samples by expression data quality as described above, the analysis included the following numbers in Experiment B: day 0: BCG n=17/control n=15; day 1: n=16/14; day 8: n=13/12; day 21: n=12/11. In Experiment C, all 12 animals were included in all stimulation conditions except for one (control pig #12) missing in the LPS stimulation.

### Cytokine quantification by ELISA (Experiment B)

In Experiment B, IL-6 and TNF were quantified from harvested supernatants using commercial porcine ELISA kits (DuoSet ELISA, R&D Systems); IFN-γ was analyzed using an in-house ELISA protocol (15).

### Statistical analysis

Data were analyzed using GraphPad Prism 6, StataMP, version 12, or SAS Enterprise Guide version 7.4.

Kruskal-Wallis was used for non-normally distributed non-paired samples and Wilcoxon sign-ranked test for non-normally distributed paired samples. In the gene expression study in Experiment B, the control and vaccinated groups were compared using unequal variances t-test. Additionally, gene response data from Experiments B and C were analyzed in multivariate models using Discriminant Analysis of Principal Components (16), in which the multivariable data consisting of numerous response variables (the combinations of stimulant and gene response) are reduced by Principal Component Analysis to fewer linear and uncorrelated variables. These canonical variables then serve as input variables in Discriminant Analysis (*proc discrim* in SAS). The discriminant analysis can test the probability of correct classification of vaccinated and unvaccinated individuals. Since we did not have a sample of adequate size to split into a training and test set, we used the *crossvalidate* and *posterr* options in SAS which classifies each observation by using a discriminant function computed from all the other observations (SAS). The misclassification rate of vaccination status is returned by the model for evaluation.

In Experiment C, LPS stimulation was lacking from one animal, and the LPS stimulation was therefore omitted from the Principal Component Analysis and Discriminant Analysis, as this method cannot handle missing values. In an alternative analysis, the missing values from this single animal were replaced by the median of each gene of the other pigs in the same treatment group (Sauton-treated, LPS-stimulated), whereby the LPS-stimulated samples could be included in the model.

### Ethics

Ethical approval for the experiments was obtained from the Danish Animal Experiments Inspectorate (case no. 2015−15−0201−00520) and the local university animal ethics committee at the Technical University of Denmark. The animal experiments took place at the research facilities of the Technical University of Denmark in 2015 and 2016.

## Results

### Safety of BCG

In total, nine piglets (BCG: 6/31; Sauton: 3/15) died or were euthanized due to events considered unrelated to the immunization in the first weeks after birth in Experiments A and B. One piglet (1/14) in Experiment A was found crushed under the sow nine days after BCG vaccination. In Experiment B, three BCG-vaccinated piglets were found dead, and two BCG-vaccinated and three Sauton-treated piglets were euthanized due to severe weakness, of which seven died within the first 7 days (**Table 1**). The causes of death or euthanasia were general weakness or mechanical conditions and did not indicate adverse events related to the BCG vaccine. No animals died prematurely in Experiment C.

**Table 1:** Deaths in the low-dose BCG study (Experiment B).

Died or euthanized after vaccination (day 0)				
Pig #	Vaccine	Sex	Day since enrolment	Cause
22	BCG	female	1	crushed
12	BCG	female	1	unknown
30	BCG	male	6	lame, euthanized
24	BCG	male	6	unknown, weak
11	BCG	male	16	lame, euthanized
29	sauton	male	1	weak, euthanized
19	sauton	male	3	euthanized
4	sauton	male	7	weak, euthanized

In total, n=17 BCG-vaccinated and n=15 Sauton-treated animals.

No visible or palpable reactions were observed at the vaccination site after the neonatal BCG dose (Experiments A and B), whereas 3/6 BCG-vaccinated pigs in the high BCG dose Experiment (Experiment C) had palpable reactions at the injection site 24 days after vaccination. Additionally, 2/6 had mild skin redness but no palpable reaction (**Supplementary Figure 1**). No pigs developed ulcers or scars.

**Figure 2:**
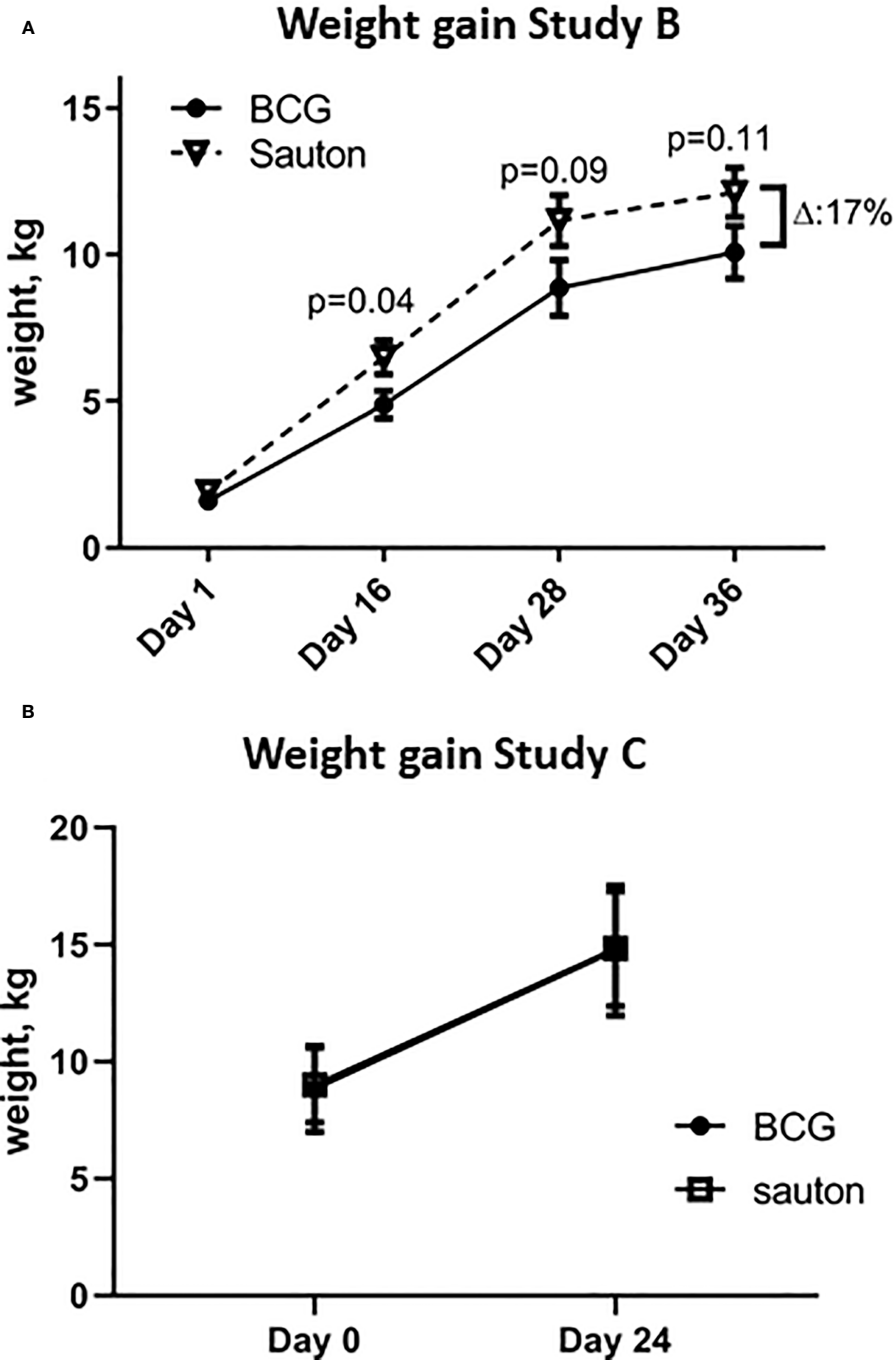
Weight gain of the animals. **(A)**. In Experiment B, the weight from day 1 to day 36 of enrolment and randomization (BCG or Sauton) of 32 piglets. Statistical test comparing BCG and Sauton-treated pigs for each time point using Kruskal-Wallis with standard error of the mean. Day 1 (BCG/Sauton): n=16/14; day 16: n=13/12; day 28: n=12/12; day 36: n=12/12. **(B)**. In Experiment C, weight from day 0 to day 24 of enrolment and randomization (BCG or Sauton) of 12 female pigs aged 5 weeks.

In Experiment B, body weights of the pigs allocated to BCG vaccination were 15% lower than the Sauton group one day after randomization (no measure of weight available before vaccination). BCG-vaccinated pigs remained lighter throughout the study, with the relatively largest difference of 25% (p=0.04) observed on day 16 (**Supplementary Table 2** and **Figure 2A**). On day 36, the BCG-vaccinated pigs were 17% (p=0.11) lighter than the Sauton-treated pigs.

Weight gain in Experiment C from randomization to 24 days after vaccination was not different between BCG and Sauton-treated pigs (**Figure 2B**).

**Figure 3:**
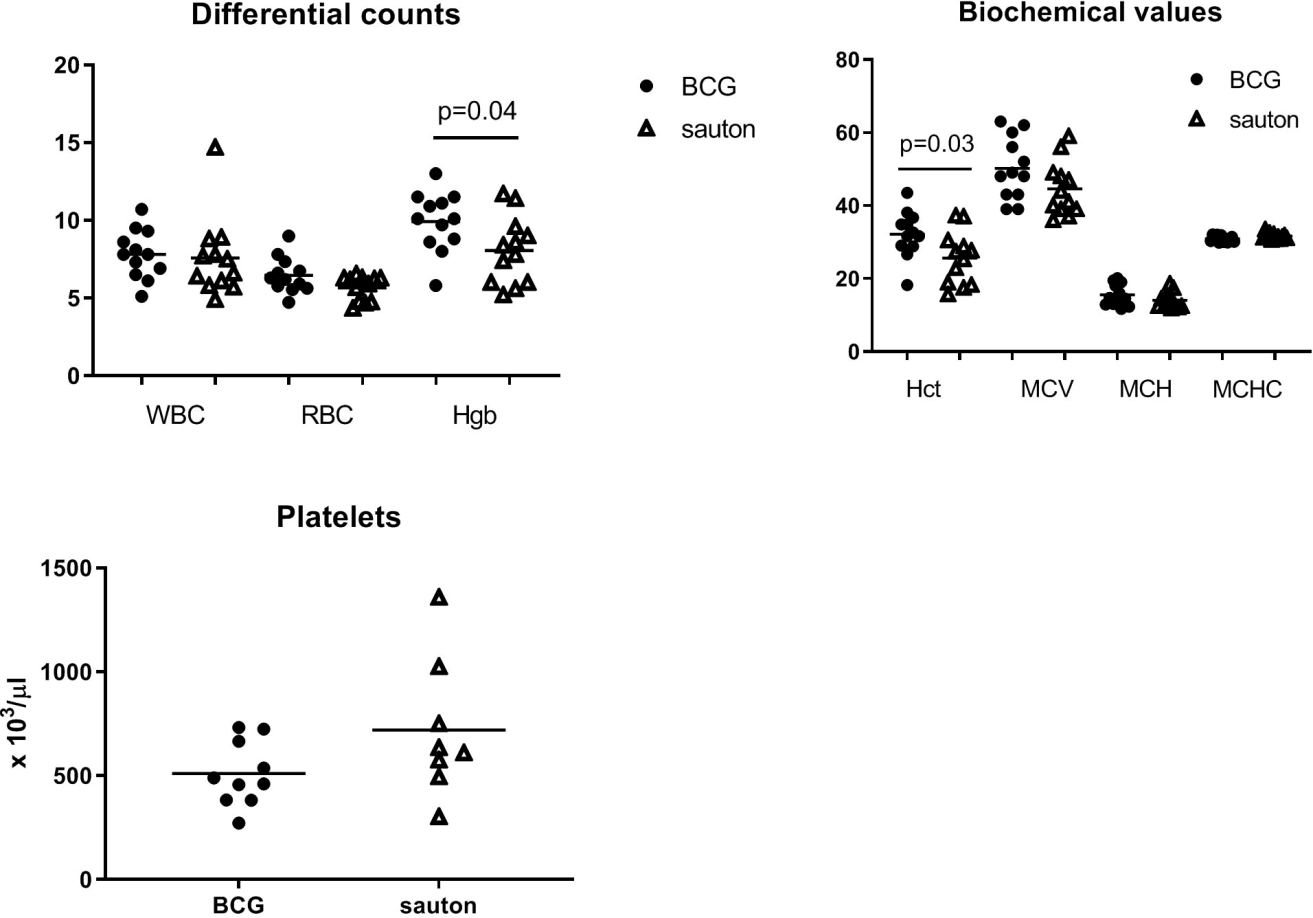
Experiment B: Hematological analysis of venous blood collected on day 30 of randomization. White blood cell counts (WBC, x 1000/μl); red blood cell counts (RBC, x 106/μl); hemoglobin concentrations (Hgb, g/dl); mean corpuscular hemoglobin (MCH, pg); hematocrit (Hct, %); mean corpuscular volume (MCV, femtoliter); mean corpuscular hemoglobin concentration (MCHC, g/dl); platelet (x 1000/μl). Statistical test comparing BCG and Sauton-treated pigs using Student’s t-test. BCG: n=12; Sauton: n=12, except for platelets: BCG: n=10; Sauton: n=8 (due to technical errors).

In Experiment B on day 30, hemoglobin and hematocrit concentrations were higher in the BCG-vaccinated pigs (p=0.04 and p=0.03, respectively) (**Figure 3**). Whole blood cell counts were not different.

### BCG persistence in tissues

In Experiment A, 20 weeks after vaccination, two of 13 pigs were culture-positive for BCG in the lateral retropharyngeal lymph node draining the vaccination site. PCR confirmed the presence of BCG. All other specimens were culture-negative and PCR-negative and there was no visible local reaction at the injection site in any of the pigs on week 20.

### *Ex vivo* whole blood gene expression

In Experiment B, the mRNA expression of 49 gene sequences was analyzed from whole blood before vaccination (day 0) and on days 1, 8, and 21 (**Supplementary Table 3**). Four genes of interest (retinoic acid-inducible gene I (RIG-I), Melanoma Differentiation-Associated protein 5 (MDA5), macrophage colony-stimulating factor 1 (CSF1), and swine leukocyte antigen DR-beta 1 (SLA-DRb)), were marginally differentially regulated between BCG and Sauton treatments at one time-point after randomization. However, none of these results sustained correction for multiple testing (**Supplementary Figure 2**).

For the remaining 44 gene transcripts tested, there was no difference between BCG-vaccinated and control. Moreover, in the multivariate analysis, using principal component analysis to reduce the data dimension, BCG-vaccinated and control animals did not segregate at any time point (data not shown). In the discriminant cross-validation analysis, the classification error rate of BCG vaccination remained high, around random annotation. Including sex as a categorizing factor together with BCG did not affect the results (data not shown).

### *In vitro* cytokine responses

**Figure 4:**
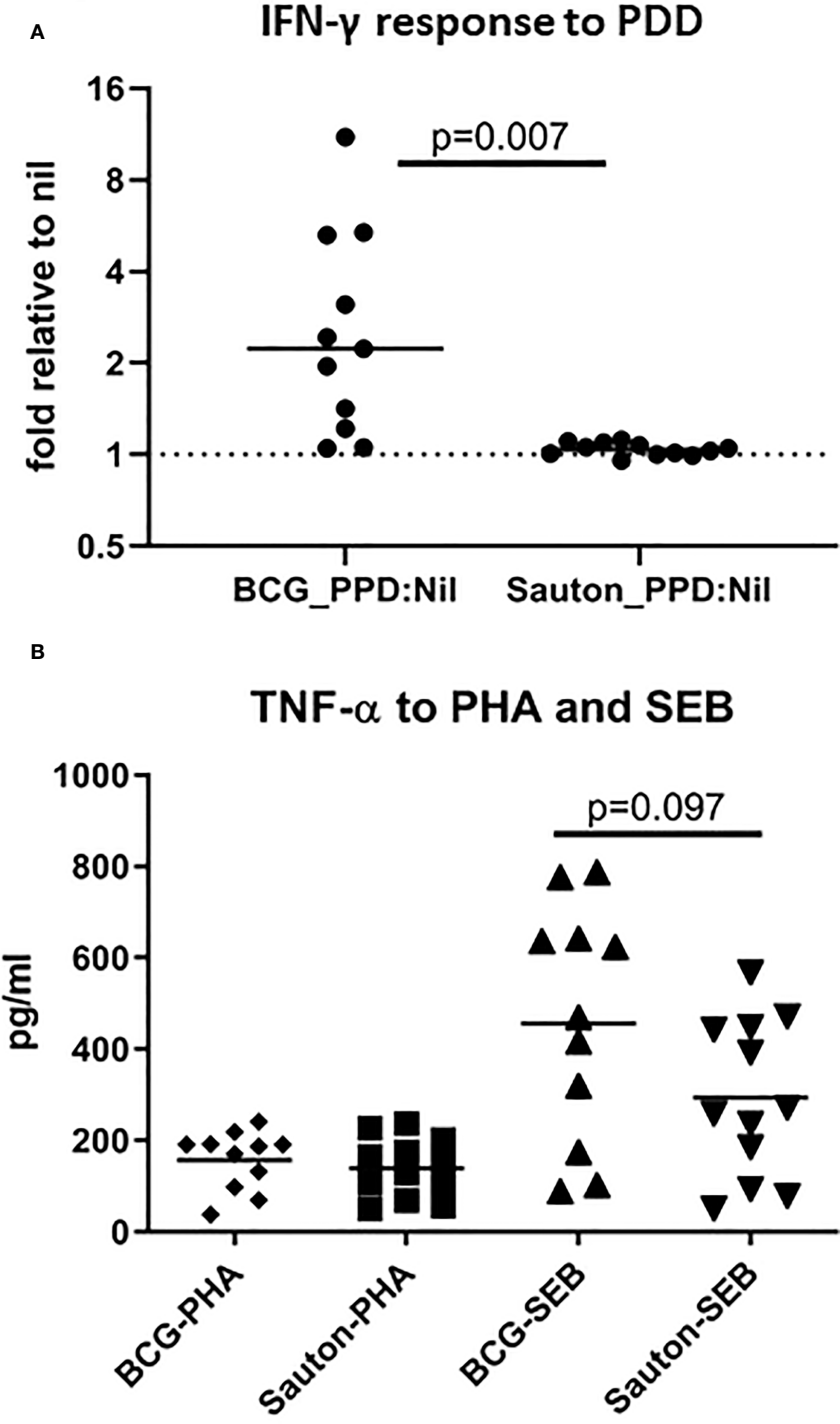
Experiment B: *In vitro* IFN-γ responses to PPD **(A)** and TNF responses to PHA or SEB **(B)**. On day 28 after randomization, undiluted whole blood was stimulated with 10 ug/ml PPD_Mtb_ or 1 ug/ml Staphylococcus enterotoxin B (SEB) or 2ug/ml phytohaemagglutinin (PHA) (including a control) for 22h, and IFN-γ and TNF were measured from harvested supernatants using ELISA. In A, the level of IFN-γ in PPD-stimulated blood is relative to the control sample (fold change over unstimulated control). The horizontal bar represents the median. Statistical test using Kruskal-Wallis. BCG n=11; Sauton n=12.

In Experiment B, 28 days after vaccination, IFN-γ responses to overnight PPD stimulation in whole blood cultures were, on average, higher in the BCG-vaccinated pigs than in control pigs (p=0.007). However, 4/11 BCG-vaccinated pigs were low responders (less than twice the nil level) (**Figure 4A**). IFN-γ responses to the positive SEB did not differ between BCG-vaccinated and control animals (data not shown). TNF responses to SEB tended to be higher (p=0.097) in BCG-vaccinated pigs, which was not seen for TNF responses to PHA (**Figure 4B**). IL-6 responses to SEB, LPS, or Pam3CSK4 were generally low compared with the nil sample with a stimulation index (stimulation to nil ratio) ranging from 1.4 for SEB, 2.0 for LPS, and 3.0 for PamCSK4. Overall, TNF and IL-6 responses to LPS or Pam3CSK4 were not different between BCG and control animals (data not shown).

**Figure 5:**
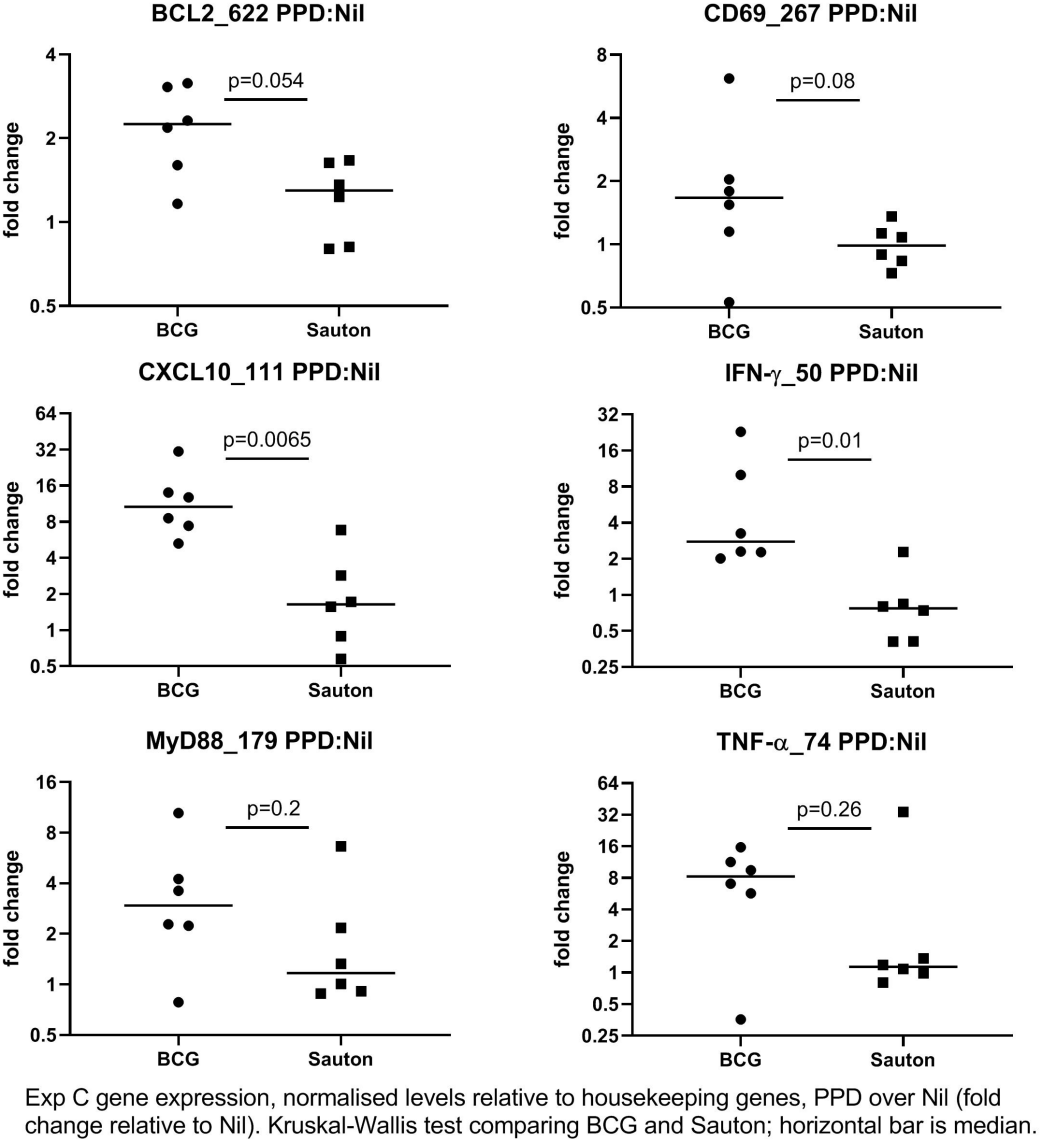
Experiment C: *In vitro* gene expression on day 24 after randomization to high-dose BCG or Sauton in PBMC stimulated with PPD (10 ug/ml), normalized to housekeeping genes, and expression levels presented relative to control (unstimulated PBMC). The horizontal bar represents the median. Statistical test using Kruskal-Wallis. BCG: n=6, Sauton: n=6.

**Figure 6:**
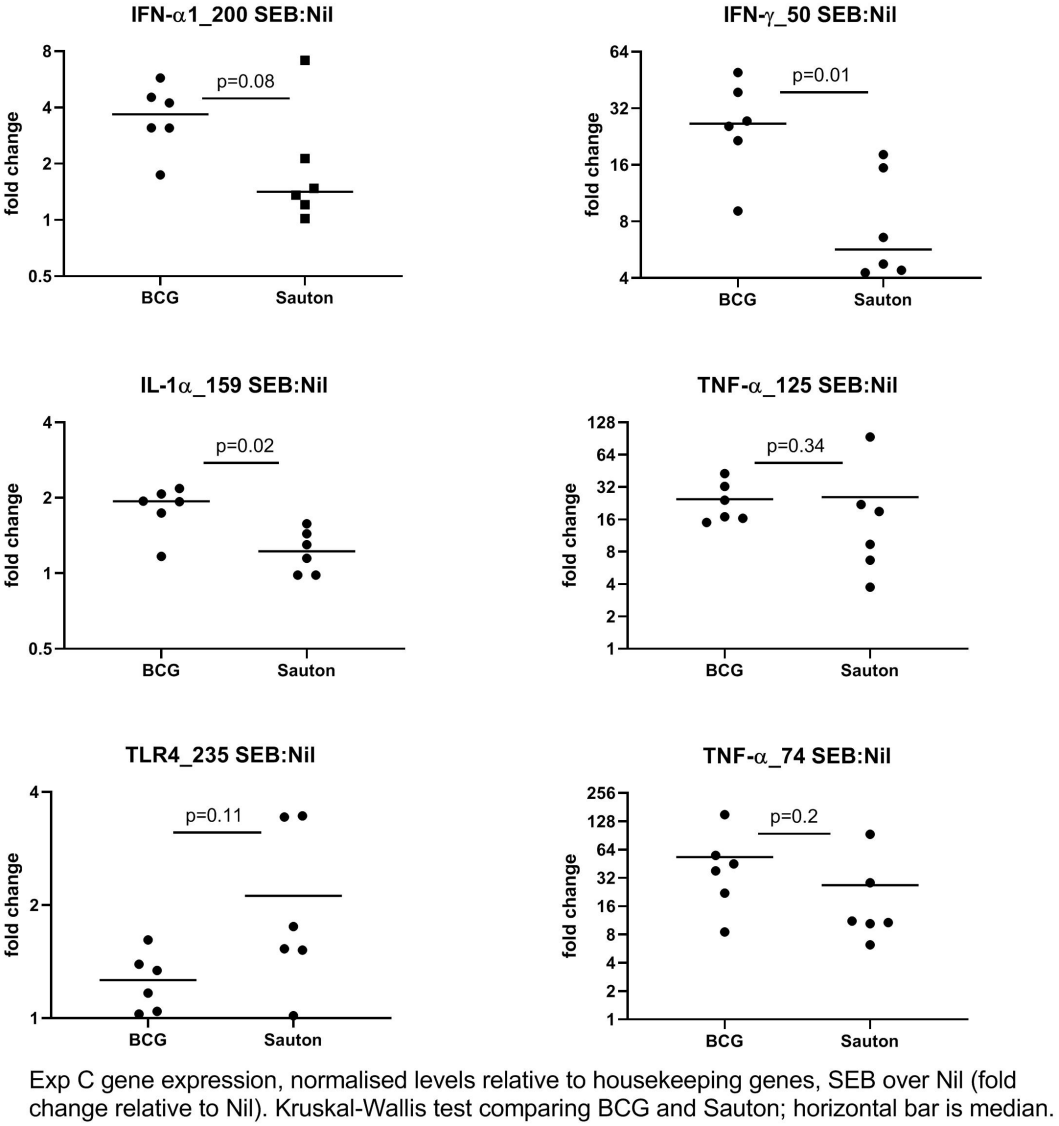
Experiment C: *In vitro* gene expression on day 24 after randomization to high-dose BCG or Sauton in PBMC stimulated with SEB (1 ug/ml), normalized to housekeeping genes, and expression levels presented relative to control (unstimulated PBMC). The horizontal bar represents the median. Statistical test using Kruskal-Wallis. BCG: n=6, Sauton: n=6.

In Experiment C, 24 days after vaccination, the expression of 52 immune-related genes from *in vitro* stimulated PBMC was measured using qPCR (**Supplementary Table 4**). Overall, gene expression levels in stimulations were relatively low compared to the medium-only controls, indicating suboptimal culture conditions, probably due to low stimulation doses. IFN-γ responses to PPD were higher than background (the medium only culture) in BCG-vaccinated pigs but not in control pigs, indicating specific immunogenicity of the BCG vaccination (**Figure 5**), as also noted for whole blood stimulations in Experiment B. For a few transcripts, responses to SEB were higher in BCG-vaccinated compared to controls, significantly so for the genes encoding the cytokines IFN-γ and IL-1α (**Figure 6**).

After principal component analysis, the 208 response variables in Experiment C (a combination of 52 gene transcripts and 4 culture stimulants including nil) were reduced to seven principal components. Next, we submitted all gene expression variables to PCA, selecting three principal components, which then served as input variables to the discriminant analysis to test if the composite gene expression data contained a signal of BCG vaccination that enabled the model to assign vaccination grouping of the 12 animals correctly. The cross-validation analysis found a total error rate of 42% (5/12) in assigning the 12 samples to either BCG or control groups, which is only slightly better than random. Including PPD response transcripts slightly improved the performance to an error rate of 33% (4/12). Imputing the missing values and including the LPS-stimulated gene responses did not improve the precision.

#### *In vitro* cytokines responses by sex

Stratifying responses by sex, BCG tended to have a larger increasing effect on IFN-γ responses to PPD in males than in females (**Supplementary Figure 3**). Also, the TNF responses to SEB were increased in BCG-vaccinated vs. Sauton-treated males (p=0.04) but not in females. A similar, although non-significant, tendency was seen for TNF responses to PHA and LPS (**Supplementary Figure 4**). In contrast, the IL-6 response to Pam3CSK4 was increased in BCG vs. control in females (p=0.04) but not in males (**Supplementary Figure 5**). The same overall tendency of higher IL-6 responses in BCG-vaccinated vs. Sauton-treated females but lower IL-6 responses in BCG-vaccinated vs. Sauton-treated males was also indicated in SEB and LPS stimulations and the nil sample (**Supplementary Figure 5**).

### Heterologous infections

**Figure 7:**
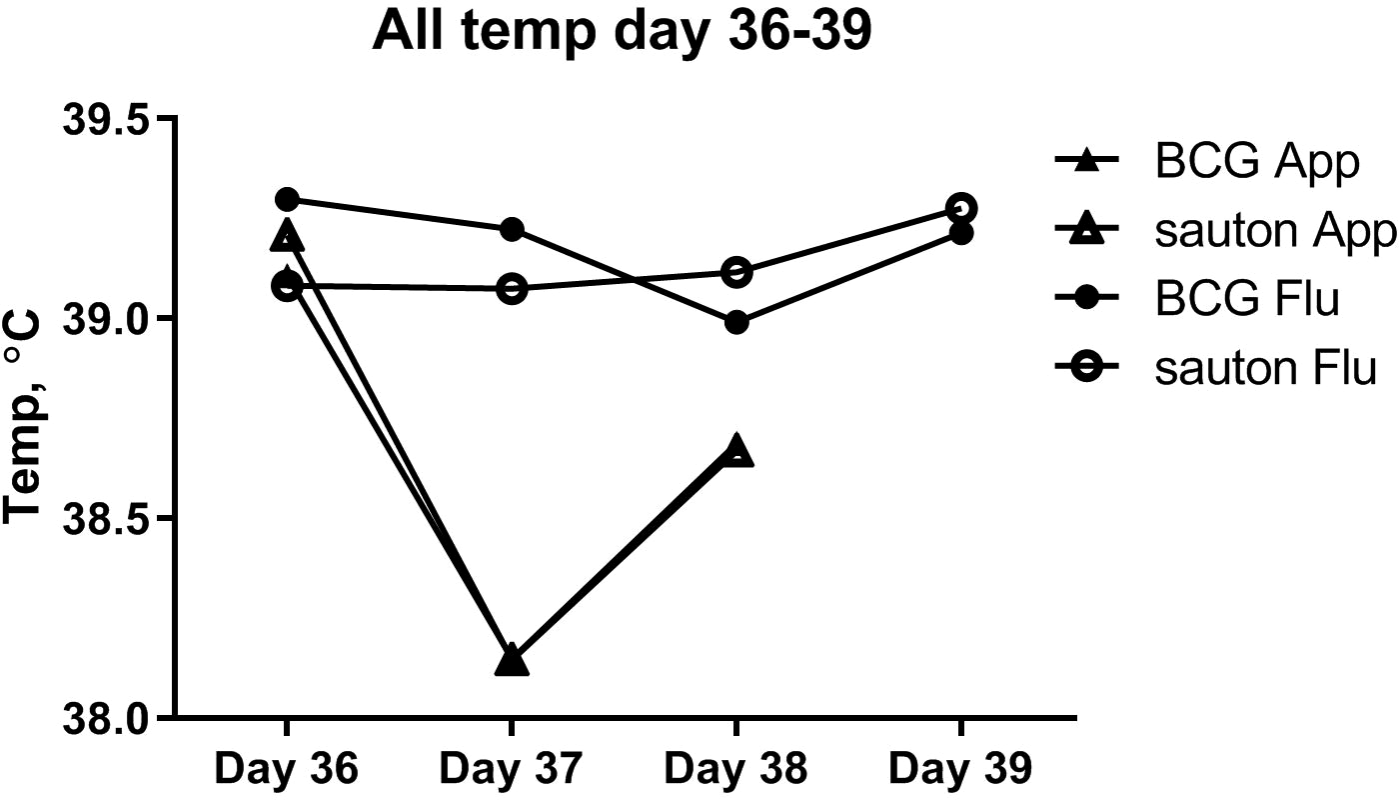
Experiment B: Temperature (rectal) during the infectious challenge. On day 36 of enrolment and randomization, animals were inoculated with *Actinobacillus pleuroneumoniae* (App) or influenza (Flu), with six animals in either treatment group.

There was overall no difference between the BCG-vaccinated and Sauton-treated pigs in rectal temperature responses to the *A. pleuropneumoniae* or influenza infections (**Figure 7**). One day after *A. pleuropneumoniae* inoculation, in both the BCG-vaccinated and Sauton-treated groups, there was a transient drop of approximately 1°C, whereas, after influenza inoculation, the temperature remained stable.

At necropsy after *A. pleuropneumoniae* inoculation, the lungs were unremarkable or showed postmortal lesions in eight pigs (BCG: n=3; Sauton: n=5). Minor lesions of consolidated lung tissue in the cranial lobes, consistent with chronic lobular bronchopneumonia, were present in three pigs (BCG: n=3). The lesions were uncharacteristic of *A. pleuropneumoniae*, but could be caused by other respiratory pathogens, e.g., *Mycoplasma* spp. One pig (BCG) had small processes of consolidated hemorrhagic lung tissue in the diaphragmatic and cranial lung lobes; though the lesions were not classic to *A. pleuropneumoniae* infection, this could not be ruled out on a macroscopical basis.

Among the influenza-inoculated pigs, the lungs were unremarkable or with postmortal lesions in nine pigs (BCG: n=6; Sauton: n=3). Mild chronic pleuritis was observed in one Sauton-treated pig. Two pigs (Sauton: n=2) showed minor lesions of consolidated lung tissue without exudation in the cranial lobes; though not typical for influenza infection this could not be excluded, however other microbes, e.g., *Mycoplasma* spp. could be responsible.

Based on RT-qPCR of the influenza matrix gene, viral load of the upper airways peaked at two days after infection but with no differences in virus titers between the BCG and Sauton groups (**Supplementary Figure 6**). No *A. pleuropneumoniae* could be cultured from any of the liver, spleen, or lung samples (data not shown).

**Figure 8:**
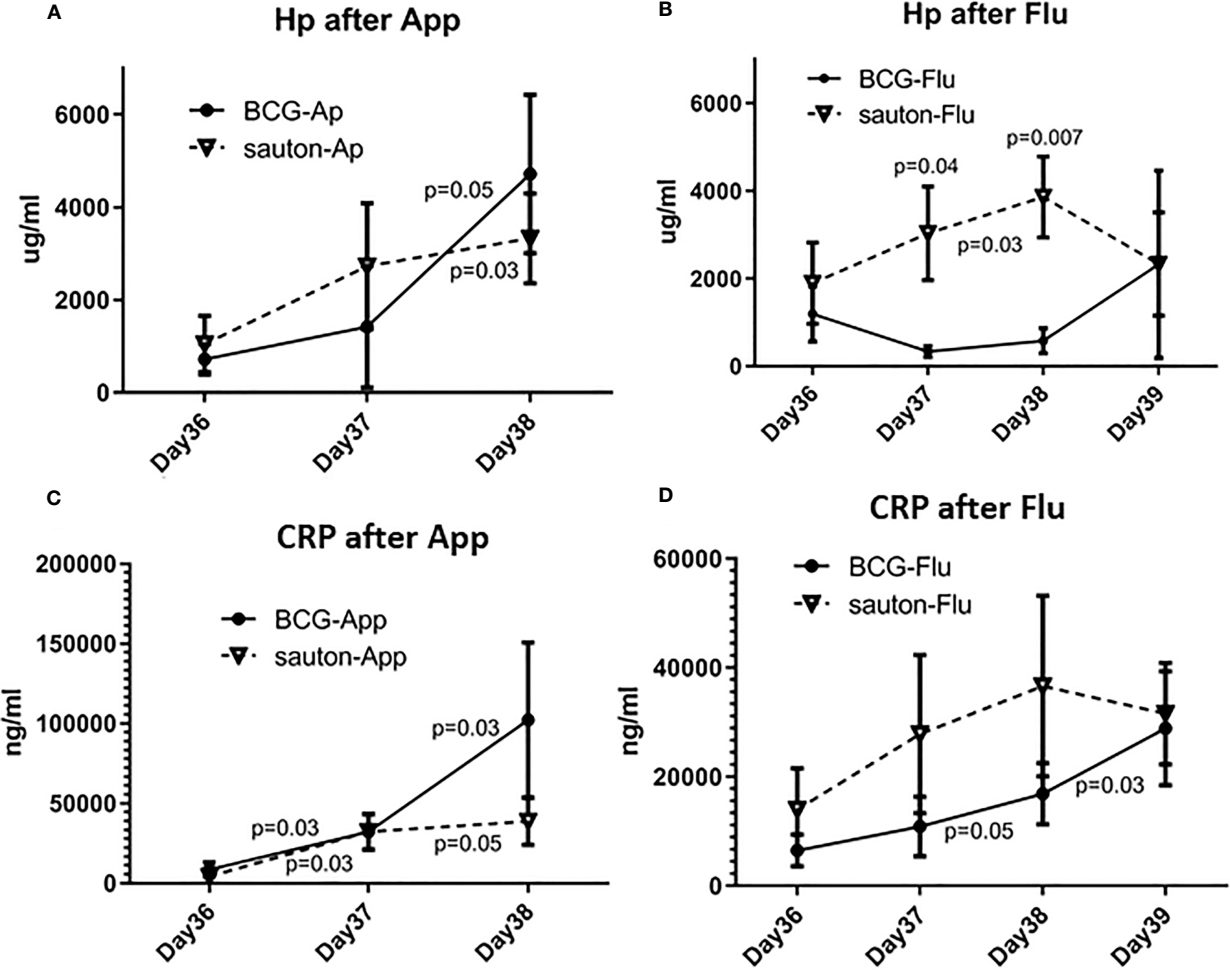
Experiment B: Acute phase reactants during the infectious challenge. On day 36 of enrolment and randomization, animals were inoculated with *Actinobacillus pleuroneumoniae* (App, A+C) or influenza (Flu, B+D), with six animals in either treatment group. Serum concentrations of Haptoglobin (Hp, A+B) and C-reactive protein (CRP, C+D) were measured with ELISA. Test of effect of infection within treatment group using Wilcoxon signed-rank test with day 36 as the comparator, with p<0.05 placed between sample days. Test of difference between vaccination groups by day of sampling using Kruskal-Wallis test, with p<0.05 placed above the day of the sample. The error bar is the standard error of the mean.

There was no difference between BCG-vaccinated and Sauton-treated pigs with respect to serum haptoglobin or CRP on the day of infectious challenge (day 36 after immunization). After *A. pleuropneumoniae* infection, serum haptoglobin concentrations increased in both the BCG and Sauton groups, with the highest levels measured two days after infection (**Figure 8A**), with no difference between the BCG and Sauton groups. After influenza infection, however, a haptoglobin response was only seen in the Sauton group, peaking at two days after infection, whereas BCG-vaccinated pigs did not show a haptoglobin response after the influenza challenge (**Figure 8B**). Likewise, serum CRP concentrations were increased after the influenza challenge, however, the response was attenuated or delayed in BCG-vaccinated pigs two days after inoculation (**Figure 8D**). No statistically significant difference in serum CRP concentrations was seen between BCG-vaccinated and Sauton-treated pigs after *A. pleuropneumoniae* challenge (**Figure 8C**).

## Discussion

In the present study, we investigated if BCG could improve resistance to heterologous infection in pigs, if BCG vaccination affected innate immunological responses, and if live BCG could be recovered from pigs up to 20 weeks after vaccination.

First, the immunological responses of neonatal pigs receiving an infant BCG vaccine dose, as well as 15-20 times the standard dose, were relatively weak, although a specific IFN-γ response to PPD stimulation was detected. Hence, the BCG vaccine-induced limited cell-mediated immune responses in our pig model.

Second, in contrast to humans, neonatal pigs receiving an infant dose of BCG did not develop local skin reactions such as abscesses or ulcers, which are usually markers of successful BCG immunization in humans (17). Only after the high-dose BCG (30-40 times the human infant dose or 15-20 times the standard human dose) could local inflammation be observed, but none of the pigs developed an ulcer or a scar.

Third, live BCG bacteria were recovered from the draining lymph node 20 weeks post-vaccination in 2/13 of the vaccinated animals, suggesting that the relatively weak immunological effects of BCG we observed in the pigs cannot be explained by rapid total clearance of the BCG bacteria after immunization.

Fourth, the acute phase protein (haptoglobin and CRP) response to mild influenza virus infection was reduced or delayed in BCG-vaccinated compared to control pigs. To our knowledge, this is the first time an attenuating effect of BCG on subsequent acute phase responses to influenza infection has been reported. Noteworthy, this effect was not seen after infection with the extracellular bacterial pathogen *A. pleuropneumoniae*, and no other parameters showed any difference between the BCG-vaccinated vs. Sauton-treated groups.

A brief summary of the major findings is presented in **Table 2**.

**Table 2:** Summary of major findings in the three studies (Experiments A, B, and C).

	Exp A	Exp B	Exp C
**Age at vaccination**	3 days	2-3 days	5 weeks
**Dose of BCG**	Infant dose	Infant dose	30-40 times infant dose
**Local reaction**	No scar	No scar	No scar, minor redness
**Persistence of live BCG**	2/13 with culture positive lymph nodes	N/A	N/A
**Immune responses specific**	N/A	Increased IFN-γ response to PPD	Increased IFN-γ response to PPD
**Immune responses non-specific**	N/A	Ex vivo gene expression overall not differential, with minor exceptions (RIG-I, MDA5, CSF1, and SLA-DRB). Responses to Pam3CSK, LPS or SEB not significantly different	Upregulated IFN-γ and IL1ra to SEB stimulation
**Heterologous challenge**	N/A	No effect on clinical responses to *A. pleuropneumoniae* challenge, whereas acute phase responses were lower or reduced after influenza challenge. No difference in nasal viral load.	N/A

N/A, not analyzed.

### Comparison with previous studies

#### BCG persistence

Studies on the persistence and biodistribution of BCG after immunization have been conducted in various species with very heterogeneous designs (animal species, dose, BCG strain, route of administration, and detection technique). The findings range from no detectable BCG one month after vaccination to BCG still detectable in tissues more than a year after vaccination (18). The very few studies in pigs or wild boars (*Sus scrofa*) find no live BCG in the tissues. From six wild Eurasian boars orally administered live BCG (15–30 oral baits containing 1×10^5^ CFU BCG per bait), no BCG was detected by culture of oropharyngeal tonsil or mandibular, parotid, retropharyngeal, tracheobronchial, mediastinal, mesenteric and hepatic lymph nodes, spleen, liver, or kidney 300 days after administration (19). A similar study detected no BCG in any tissues or from nasal, oral, and rectal swabs from wild boars 175-300 days after oral BCG administration (18). In wild boars given approximately 4.5 × 10^6^ CFU of BCG Denmark as oral bait, no BCG could be cultivated from lymph nodes, muscle tissue, or inner organs 1, 3, 6, or 9 months after vaccination (20). The present study is the first to investigate BCG persistence in pigs after i.d. administration of what corresponds to an infant dose of BCG. In comparison, a single study has been conducted in 15 human adults, in which BCG could not be recovered from bone marrow aspirates 90 days after BCG i.d. vaccination, despite robust induction of trained immunity (21).

#### Immunogenicity of the BCG vaccine in pigs

There is strong evidence of beneficial non-specific effects of BCG, including reduced all-cause mortality in human children (2). Mechanistic explanations for the non-specific effects of BCG may include innate training of monocytes and emergency granulopoiesis demonstrated *in vivo* in human adults (22) and human and murine newborns (23), respectively. Previous studies, however, are conflicting on the magnitude of the non-specific effects of BCG on inflammatory responses in human newborns (24). For example, in Danish newborns, there was no evidence of increased innate responses until one year after BCG immunization (25), whereas in a comparable study in human low-weight infants in Guinea-Bissau, early BCG vaccination induced increased responsiveness in whole blood cultures of IL-1β, IL-6, TNF, and IFN-γ to the innate agonist Pam3CSK4 four weeks after vaccination, with TNF responses being 29% increased, compared with late BCG; the *M. tuberculosis*-specific PPD responses of IFN-γ were raised by a factor of 13 (26).

Despite some differences in study design, including a much smaller sample size, it is evident that we did not observe such magnitude of non-specific effects of BCG in piglets. A potential caveat to the interpretation of the innate responses may have been that the *in vitro* conditions of LPS and Pam3CKS4 overall induced relatively low cytokine responses over the non-stimulated (nil) culture samples. Pigs are less sensitive to endotoxins compared with humans (27). A recent study of porcine monocytes used a 10 times stronger concentration of LPS (100ng/ml) for *in vitro* stimulations (28).

In an important proof-of-concept experiment, Byrne et al. showed that BCG Denmark could train porcine monocytes *in vitro* to increase responses of IL-1β and TNF against subsequent LPS stimulation (28). Trained immunity of monocytes by BCG has previously been demonstrated in humans and mice (22). Trained immunity of BCG as *in vivo* vaccination has not been shown directly in pigs.

The minor innate cytokine responses and somewhat more robust SEB-stimulated responses, together with the *M. tuberculosis*-specific IFN-γ responses, suggests that the effect of BCG in our model was not a myeloid progenitor biased induction, but rather an increase in specific Th1 lymphocytes.

There is evidence of specific immune responses to BCG in porcine species, although from only a few studies. In a controlled study of neonatal minipigs receiving a human infant dose of i.d. BCG Vaccine SSI 2 days after birth, there was no difference in peripheral CD4+ or CD8+ T cell or monocyte frequencies up to 12 weeks after BCG. After the *M. tuberculosis* challenge, however, monocyte numbers increased more in BCG-vaccinated pigs, and the number of *M. tuberculosis* culture-positive animals and bacterial load were reduced, indicating some specific protective efficacy of BCG (29). BCG ingested from oral bait was also protective against *M. bovis* challenge in wild boar (30). Lee et al. administered BCG Vaccine SSI 10^7^ CFU subcutaneously (s.c.) to 3-5 weeks old male mixed-breed domesticated pigs, and although the authors did not report adverse events or clinical outcomes, the study demonstrated that BCG was immunogenic with *M. bovis*-specific IFN-γ responses and lymphoproliferation 3-4 weeks after BCG immunization, including CD4+ T cells and γδ T cells (31). Increased *in vitro* killing activity against a human cancer cell line was observed in γδ T cells from male pigs of mixed-breed vaccinated i.d. with 6x10^6^ CFU BCG Vaccine SSI at 3 weeks of age. On day 14 after BCG vaccination, there was additionally an increased γδ T cell killing of BCG-infected monocytes compared to killing by CD8+ T cells, while on day 21 after BCG, the CD8+ T cell cytolytic activity was comparable to the γδ T cell killing (32). Importantly, pigs have high numbers of circulating γδ T cells, especially at a young age (33). It could be speculated whether the activated γδ T cells more effectively clear phagocytic cells bearing BCG compared with, e.g., humans, thereby blunting the innate training effect *in vivo*.

In 14 young wild boars, less than one year old, of which half received 0.1ml BCG Vaccine SSI i.d., genetic expression of genes encoding IFN-γ, C3, RANTES, and IL-4 from non-stimulated PBMC was analyzed 0, 5, 13, and 21 weeks after BCG. IFN-γ and C3 were transiently upregulated in the BCG recipients at 5 weeks, whereas IL-4 and RANTES were transiently upregulated at 13 weeks (34). In our study, gene expression of IFN-γ and IL-4 was analyzed in non-stimulated whole blood at 1 and 3 weeks after immunization, with no difference between BCG and control.

A long-lasting immunological effect of heat-inactivated wild-type *M. bovis* was observed 10 weeks after an oral booster dose. Vaccinated boars had increased serum levels of IL-1β and IL-6 and increased mRNA expression in resting PBMC of NLRP3 and MyD88 (35).

A highly variable and, for some animals, delayed immunological response to BCG was seen in 10-week-old pigs immunized i.d. with BCG Vaccine SSI 2x10^6^ CFU, in which IFN-γ responses to *in vitro* PPD stimulation were evaluated on days 0, 20, 41, and 118 after vaccination; at day 20, only 2/6 pigs had positive PPD IFN-γ responses, but all were positive for lymphoproliferation at day 118 (36). Taken together, the above evidence indicates a delayed immunological response to BCG in pigs, including a delayed maturation of the T-cell specific and non-specific responses, which would not have been captured by the present experimental design with neonatal vaccination and a relatively short follow-up period.

#### BCG dose and route of administration

In the present study, dose optimization of BCG was not performed and has, to our knowledge, not been conducted in pigs *in vivo*. We decided to use a human infant dose for the first studies (Experiments A and B) to mimic human conditions since the weight of the neonatal pig is comparable to the human newborn. Prompted by the relatively low specific and non-specific immunological effects of the infant dose BCG (Experiment B), we increased the dose 30-40 times (equaling 15-20 times the standard human dose) in Experiment C, as we speculated that an infant dose was eliminated too rapidly in the pigs. Murine experiments have generally used a much larger dose of BCG by body weight. For instance, Kleinnijenhuis et al. applied a 20-time infant dose of BCG to SCID mice subsequently challenged with lethal *Candida albicans*. Considering the significantly smaller size of the mice (appr. 22 g), the weight-indexed dose difference between the murine study and the present porcine study (appr. 1 kg) is accordingly larger. In the murine study, BCG was highly protective against *C. albicans* infection (22). A study in human adults found no differences between the standard dose BCG and twice that dose on trained immunity, although cytokine response capacity by NK cells may have been slightly larger after the double-dose BCG (37). A direct analysis of the BCG dose effect was not possible as the assessment methods of the immunological effects were different in Experiment B and Experiment C, the animals were older in Experiment C, and Experiment C only used females. In contrast, Experiment B was a mix of males and females.

Importantly, BCG was applied intradermally as per standard in humans. A Danish multicenter clinical trial of neonatal BCG vaccination has shown that injection technique is a determinant of developing a scar (38), potentially also affecting the immunogenicity. A meta-analysis compiling evidence from human studies found that among BCG-vaccinated children, developing a BCG scar vs. not developing a BCG scar is associated with 39% (24-49%) lower all-cause mortality (17). Previous immunization experiments in pigs, however, indicate that BCG may induce immunologic responses via the peroral (34), intradermal (29, 32), and subcutaneous route (31). The striking absence of abscesses, ulcers, or scars in our experiments may result from fundamental local differences in tissue repair mechanisms or immunological properties of the skin of pigs compared with humans. In other experiments conducted after the present study, a complete resolution of intradermal injection with live *Staphylococcus aureus* was observed when injected on the back or the side of the pig. In contrast, i.d. injection in the inner thigh resulted in purulent inflammatory lesions more comparable to *S. aureus* skin lesions in humans (G Jungersen, unpublished studies). Hence, route and injection site may be considered in future studies of BCG using the pig as a model.

Route of administration may influence how BCG is distributed and localized in host tissues. Interestingly, comparing intravenous (i.v.) vs s.c. administration in mice, Kaufmann et al. reported that BCG could be recovered in the bone marrow up to 7 months after vaccination using the i.v. route, whereas persistence of BCG was much reduced and shorter using the s.c. route. The load of BCG recovered from the bone marrow correlated with the increase of hematopoietic progenitor cells, and overall, the i.v. route promoted a stronger polarization of multipotent progenitor cells towards myelopoiesis and bone-marrow derived macrophages demonstrated better protective efficacy against *M. tuberculosis* challenge compared with the s.c. route (39).

#### Heterologous protective effects of BCG

Protective effects of BCG against non-related pathogens have been demonstrated in humans (40) and in several vertebrate animal species, including mice, rabbits (41), and fish (flounders) (42); however, no such evidence has been established in pigs.

The *A. pleuropneumoniae* infectious dose was relatively weak and induced only mild clinical symptoms and no necrotic lesions that could be attributed to the infection. *A. pleuropneumoniae* is the causative agent of porcine pleuropneumonia, a rapidly evolving disease, which can be lethal within a few hours or days of acquisition. The *A. pleuropneumoniae* infection model has been used previously by our group (43, 44). In one study, fibrino hemorrhagic lesions could be observed in the pig lungs only 6h after inoculation (43). We suspect that the failure to induce macroscopic lesions was due to a suboptimal bacterial load of the inoculum. We have previously observed indications that the non-specific effects of an inactivated mycobacterial vaccine depended on the dose of *A. pleuropneumoniae* in a heterologous challenge (44), in that inactivated mycobacterial vaccine exacerbated the necrotic effects of a high-dose *A. pleuropneumoniae* infection, whereas it had ameliorating effects when pigs were infected with a low dose *A. pleuropneumoniae* (44). Interpolating those findings to the present study would suggest that the experimental conditions favored a beneficial effect of BCG vaccination, although a notable caveat would be the difference in the live vs. inactivated nature of the vaccine.

After the influenza challenge, we observed no differences in virus titers retrieved from the nostrils. A recent study in mice and hamsters found that BCG reduced lethality, viral lung load, and lung tissue damage after challenge with influenza but not SARS CoV-2 (45), indicating that the protective effect of BCG depends on the properties of the heterologous pathogen. In particular, the authors suggested that SARS hampered the BCG-elicited trained immunity of the bone-marrow residing hematopoietic stem cells, as SARS may directly target the bone marrow, in contrast to the influenza virus. Despite nasal viral loads being unaffected by BCG in pigs, there was a reduced acute phase response in BCG-vaccinated pigs after the viral challenge. A similar effect of a reduced systemic inflammatory state after BCG in response to a heterologous challenge infection was observed in human adults receiving live yellow fever vaccine 4 weeks after BCG vaccination, where BCG vaccination before yellow fever was associated with reduced day 5 viremia accompanied by reduced levels of circulating inflammatory cytokines in the peripheral blood (46). However, in a human experimental malaria model, a subgroup of volunteers receiving BCG vaccination before the malaria challenge experienced reduced malaria parasitemia, concomitant with more pronounced clinical symptoms, increased early innate responses to the malaria challenge, increased plasma inflammatory IFN-γ, granzyme B and C-reactive protein, and increased cytolytic activity of NK cells in response to *Plasmodium falciparum*, compared with non-vaccinated control subjects (47). Whether BCG ameliorates or increases the acute inflammatory responses to heterologous infection may therefore depend on the nature of the pathogen.

## Conclusion

In conclusion, we did not find a non-specific protective effect of BCG on gene expression, innate training, or the clinical outcomes of a bacterial or viral challenge infection in pigs. However, the acute phase response to the influenza infection was lowered/delayed in the BCG-treated group. This was not seen with the *A. pleuropneumoniae*-challenged pigs. Pigs developed no or significantly milder local skin reactions after BCG vaccination, contrasting the scarification seen in humans after intradermal BCG. Finally, BCG was recovered from lymph nodes at 20 weeks after BCG immunization, which further reduces the feasibility of BCG administration to slaughter pigs.

## Data availability statement

The gene expression data in this study can be found in online repositories. The names of the repository/repositories and accession number(s) can be found in the article/**Supplementary Material**.

## Ethics statement

The animal study was reviewed and approved by Danish Animal Experiments Inspectorate and Technical University of Denmark.

## Author contributions

Conceptualization: CB, KJ, GJ, LL, and PH; Methodology: KJ, GJ, CB, PH, LL, MH, KS, and ES; Laboratory investigation: KJ and MH; Veterinary supervision: GJ and MH; Data curation, software, and formal statistical analysis: KJ; Visualization: KJ; Writing- original draft: KJ; Writing, reviewing and editing: KJ, CB, PH, GJ, KS, MH, ES, and LL; Funding acquisition: CB, GJ, and KJ; Project administration: KJ. All authors contributed to the article and approved the submitted version.

## Supplementary material

Supplementary Figure 1 Photo of local reaction to high-dose BCG in Experiment C, day 24 (Pig #11).

Supplementary Figure 2
*Ex vivo* gene expression of selected genes in Experiment B. The error bar is the standard deviation.

Supplementary Figure 3
*In vitro* IFN-γ responses, by sex of the animal, in Experiment B. On day 28, undiluted whole blood was stimulated with 10 µg/ml PPD_Mtb_ or 1 µg/ml Staphylococcus enterotoxin B (SEB) or unstimulating medium (nil). The value in the two upper panels is relative to the control sample (fold change over unstimulated control). The horizontal bar represents the median. Statistical test using Kruskal-Wallis. BCG male: n=6; Sauton male: n=5; BCG female: n=5; Sauton female: n=7.

Supplementary Figure 4
*In vitro* TNF responses, by sex of the animal, in Experiment B. On day 28, undiluted whole blood was stimulated with LPS (10 ng/ml), Pam3CSK4 (1 µg/ml), SEB (1µg/ml) or PHA (2µg/ml) for 22 hours. The horizontal bar represents the median. Statistical test using Kruskal-Wallis. BCG male: n=6; Sauton male: n=5; BCG female: n=5; Sauton female: n=7.

Supplementary Figure 5
*In vitro* IL-6 responses, by sex of the animal, in Experiment B. On day 28, undiluted whole blood was stimulated with LPS (10 ng/ml), Pam3CSK4 (1 µg/ml), SEB (1µg/ml) or PHA (2µg/ml) for 22 hours. The horizontal bar represents the median. Statistical test using Kruskal-Wallis. BCG male: n=6; Sauton male: n=5; BCG female: n=5; Sauton female: n=7.

Supplementary Figure 6 Level of influenza virus RNA from nasopharyngeal swabs (Experiment B), quantified using semi-quantitative PCR immediately before (day 36) and after (days 37-39) influenza inoculation.

Supplementary Table 1 List of primers for gene expression studies in Experiments B and C.

Supplementary Table 2 Weight and temperature in Experiment B.

Supplementary Table 3 Gene expression data from Experiment B, ex vivo blood. Gene expression values were scaled relative to the lowest value within each gene sequence and log2-transformed, setting this to 1.

Supplementary Table 4 Gene expression data from Experiment C, stimulated PBMC. Gene expression values were scaled relative to the lowest value within each gene sequence, setting this to 1.

## References

[1] Higgins JP Soares-Weiser K Lopez-Lopez JA Kakourou A Chaplin K Christensen H. Association of BCG, DTP, and measles containing vaccines with childhood mortality: systematic review. Bmj (2016) 355:i5170. doi: 10.1136/bmj.i5170 27737834 PMC5063034

[2] Biering-Sorensen S Aaby P Lund N Monteiro I Jensen KJ Eriksen HB. Early BCG-Denmark and neonatal mortality among infants weighing <2500 g: a randomized controlled trial. Clin Infect Dis (2017) 65:1183–90. doi: 10.1093/cid/cix525 PMC5849087 29579158

[3] Ziogas A Netea MG. Trained immunity-related vaccines: innate immune memory and heterologous protection against infections. Trends Mol Med (2022) 28:497–512. doi: 10.1016/j.molmed.2022.03.009 35466062

[4] Brook B Harbeson DJ Shannon CP Cai B He D Ben-Othman R. BCG Vaccination-induced emergency granulopoiesis provides rapid protection from neonatal sepsis. Sci Transl Med (2020) 12:eaax4517. doi: 10.1126/scitranslmed.aax4517 32376769 PMC8008103

[5] Ramos L Lunney JK Gonzalez-Juarrero M. Neonatal and infant immunity for tuberculosis vaccine development: importance of age-matched animal models. Dis Models Mech (2020) 13:dmm045740. doi: 10.1242/dmm.045740 PMC7520460 32988990

[6] Pabst R. The pig as a model for immunology research. Cell Tissue Res (2020) 380:287–304. doi: 10.1007/s00441-020-03206-9 32356014 PMC7223737

[7] Williams GA Scott-Baird E Núñez A Salguero FJ Wood E Houghton S. The safety of BCG vaccination in cattle: results from good laboratory practice safety studies in calves and lactating cows. Heliyon (2022) 8:e12356. doi: 10.1016/j.heliyon.2022.e12356 36590473 PMC9800532

[8] Pasco ST Anguita J. Lessons from bacillus calmette-guérin: harnessing trained immunity for vaccine development. Cells (2020) 9:2109. doi: 10.3390/cells9092109 32948003 PMC7564904

[9] Trebbien R Bragstad K Larsen LE Nielsen J Botner A Heegaard PM. Genetic and biological characterisation of an avian-like H1N2 swine influenza virus generated by reassortment of circulating avian-like H1N1 and H3N2 subtypes in Denmark. Virol J (2013) 10:290. doi: 10.1186/1743-422X-10-290 24047399 PMC3851529

[10] De Vleeschauwer A Atanasova K Van Borm S van den Berg T Rasmussen TB Uttenthal Å. Comparative pathogenesis of an avian H5N2 and a swine H1N1 influenza virus in pigs. PloS One (2009) 4:e6662. doi: 10.1371/journal.pone.0006662 19684857 PMC2722722

[11] Heegaard PM Pedersen HG Jensen AL Boas U. A robust quantitative solid phase immunoassay for the acute phase protein c-reactive protein (CRP) based on cytidine 5’-diphosphocholine coupled dendrimers. J Immunol Methods (2009) 343:112–8. doi: 10.1016/j.jim.2009.02.002 19236874

[12] Sorensen NS Tegtmeier C Andresen LO Pineiro M Toussaint MJ Campbell FM. The porcine acute phase protein response to acute clinical and subclinical experimental infection with streptococcus suis. Vet Immunol Immunopathol (2006) 113:157–68. doi: 10.1016/j.vetimm.2006.04.008 16774789

[13] Starbæk SMR Andersen MR Brogaard L Spinelli A Rapson V Glud HA. Innate antiviral responses in porcine nasal mucosal explants inoculated with influenza a virus are comparable with responses in respiratory tissues after viral infection. Immunobiology (2022) 227:152192. doi: 10.1016/j.imbio.2022.152192 35255458 PMC8863374

[14] Lüthje FL Skovgaard K Jensen HE Kruse Jensen L. Pigs are useful for the molecular study of bone inflammation and regeneration in humans. Lab Anim (2018) 52:630–40. doi: 10.1177/0023677218766391 29653496

[15] Riber U Boesen HT Jakobsen JT Nguyen LT Jungersen G. Co-Incubation with IL-18 potentiates antigen-specific IFN-gamma response in a whole-blood stimulation assay for measurement of cell-mediated immune responses in pigs experimentally infected with lawsonia intracellularis. Vet Immunol Immunopathol (2011) 139:257–63. doi: 10.1016/j.vetimm.2010.09.001 20889217

[16] Jombart T Devillard S Balloux F. Discriminant analysis of principal components: a new method for the analysis of genetically structured populations. BMC Genet (2010) 11:94. doi: 10.1186/1471-2156-11-94 20950446 PMC2973851

[17] Benn CS Roth A Garly M-L. Fisker AB Schaltz-Buchholzer F Timmermann A. BCG Scarring and improved child survival: a combined analysis of studies of BCG scarring. J Intern Med (2020) 288:614–24. doi: 10.1111/joim.13084 32301189

[18] Beltrán-Beck B Romero B Sevilla IA Barasona JA Garrido JM González-Barrio D. Assessment of an oral mycobacterium bovis BCG vaccine and an inactivated m. bovis preparation for wild boar in terms of adverse reactions, vaccine strain survival, and uptake by nontarget species. Clin Vaccine Immunol (2014) 21:12–20. doi: 10.1128/CVI.00488-13 24173022 PMC3910919

[19] Ballesteros C Garrido JM Vicente J Romero B Galindo RC Minguijón E. First data on Eurasian wild boar response to oral immunization with BCG and challenge with a mycobacterium bovis field strain. Vaccine (2009) 27:6662–8. doi: 10.1016/j.vaccine.2009.08.095 19747578

[20] Nol P Robbe-Austerman S Rhyan JC McCollum MP Triantis JM Beltrán-Beck B. Determining the persistence of mycobacterium bovis bacille calmette–guerin Danish in select tissues of orally vaccinated feral swine (Sus scrofa ssp. ). Res Veterinary Sci (2016) 104:50–2. doi: 10.1016/j.rvsc.2015.11.007 26850536

[21] Cirovic B de Bree LCJ Groh L Blok BA Chan J van der Velden WJFM. BCG Vaccination in humans elicits trained immunity via the hematopoietic progenitor compartment. Cell Host Microbe (2020) 28:322–334.e5. doi: 10.1016/j.chom.2020.05.014 32544459 PMC7295478

[22] Kleinnijenhuis J Quintin J Preijers F Joosten LA Ifrim DC Saeed S. Bacille calmette-guerin induces NOD2-dependent nonspecific protection from reinfection via epigenetic reprogramming of monocytes. Proc Natl Acad Sci USA (2012) 109:17537–42. doi: 10.1073/pnas.1202870109 PMC3491454 22988082

[23] Brook B Schaltz-Buchholzer F Ben-Othman R Kollmann T Amenyogbe N. A place for neutrophils in the beneficial pathogen-agnostic effects of the BCG vaccine. Vaccine (2022) 40:1534–9. doi: 10.1016/j.vaccine.2021.03.092 PMC11688641 33863572

[24] Butkeviciute E Jones CE Smith SG. Heterologous effects of infant BCG vaccination: potential mechanisms of immunity. Future Microbiol (2018) 13:1193–208. doi: 10.2217/fmb-2018-0026 PMC6190278 30117744

[25] Nissen TN Birk NM Blok BA Arts RJW Andersen A Kjaergaard J. Bacillus calmette-guerin vaccination at birth and *in vitro* cytokine responses to non-specific stimulation. a randomized clinical trial. Eur J Clin Microbiol Infect Dis (2018) 37:29–41. doi: 10.1007/s10096-017-3097-2 28890996

[26] Jensen KJ Larsen N Biering-Sorensen S Andersen A Eriksen HB Monteiro I. Heterologous immunological effects of early BCG vaccination in low-birth-weight infants in Guinea-Bissau: a randomized-controlled trial. J Infect Dis (2015) 211:956–67. doi: 10.1093/infdis/jiu508 PMC4340366 25210141

[27] Redl H Bahrami S Schlag G Traber DL. Clinical detection of LPS and animal models of endotoxemia. Immunobiology (1993) 187:330–45. doi: 10.1016/S0171-2985(11)80348-7 8330902

[28] Byrne KA Tuggle CK Loving CL. Differential induction of innate memory in porcine monocytes by **β** -glucan or bacillus calmette-guerin. Innate Immun (2021) 27:448–60. doi: 10.1177/1753425920951607 PMC8504267 32862748

[29] Ramos L Obregon-Henao A Henao-Tamayo M Bowen R Izzo A Lunney JK. Minipigs as a neonatal animal model for tuberculosis vaccine efficacy testing. Veterinary Immunol Immunopathology (2019) 215:109884. doi: 10.1016/j.vetimm.2019.109884 31420066

[30] Gortazar C Beltrán-Beck B Garrido JM Aranaz A Sevilla IA Boadella M. Oral re-vaccination of Eurasian wild boar with mycobacterium bovis BCG yields a strong protective response against challenge with a field strain. BMC Vet Res (2014) 10:96. doi: 10.1186/1746-6148-10-96 24766746 PMC4005810

[31] Lee J Choi K Olin MR Cho SN Molitor TW. Gammadelta T cells in immunity induced by mycobacterium bovis bacillus calmette-guerin vaccination. Infection Immun (2004) 72(3):1504–11. doi: 10.1128/IAI.72.3.1504-1511.2004 PMC355996 14977956

[32] Olin MR Hwa Choi K Lee J Molitor TW. γδ T-lymphocyte cytotoxic activity against mycobacterium bovis analyzed by flow cytometry. J Immunol Methods (2005) 297:1–11. doi: 10.1016/j.jim.2004.10.002 15777926

[33] Sinkora M Butler JE. The ontogeny of the porcine immune system. DevComp Immunol (2009) 33:273–83. doi: 10.1016/j.dci.2008.07.011 PMC7103207 18762210

[34] Lastra J Galindo R Gortazar C Ruizfons F Aranaz A Delafuente J. Expression of immunoregulatory genes in peripheral blood mononuclear cells of European wild boar immunized with BCG. Veterinary Microbiol (2009) 134:334–9. doi: 10.1016/j.vetmic.2008.08.026 19095381

[35] Beltrán-Beck B de la Fuente J Garrido JM Aranaz A Sevilla I Villar M. Oral vaccination with heat inactivated mycobacterium bovis activates the complement system to protect against tuberculosis. PloS One (2014) 9:e98048. doi: 10.1371/journal.pone.0098048 24842853 PMC4026474

[36] Bruffaerts N Pedersen LE Vandermeulen G Préat V Stockhofe-Zurwieden N Huygen K. Increased b and T cell responses in m. bovis bacille calmette-guérin vaccinated pigs Co-immunized with plasmid DNA encoding a prototype tuberculosis antigen. PloS One (2015) 10:e0132288. doi: 10.1371/journal.pone.0132288 26172261 PMC4501720

[37] Debisarun PA Kilic G de Bree LCJ Pennings LJ van Ingen J Benn CS. The impact of BCG dose and revaccination on trained immunity. Clin Immunol (2023) 246:109208. doi: 10.1016/j.clim.2022.109208 36565972

[38] Jensen TM Jensen SK Birk NM Rieckmann A Hoffmann T Benn CS. Determinants of bacille calmette-guérin scarification in Danish children. Heliyon. (2021) 7:e05757. doi: 10.1016/j.heliyon.2020.e05757 33474505 PMC7803645

[39] Kaufmann E Sanz J Dunn JL Khan N Mendonca LE Pacis A. BCG Educates hematopoietic stem cells to generate protective innate immunity against tuberculosis. Cell. (2018) 172:176–90. doi: 10.1016/j.cell.2017.12.031 29328912

[40] Fritschi N Curtis N Ritz N. Bacille calmette guérin (BCG) and new TB vaccines: specific, cross-mycobacterial and off-target effects. Paediatric Respir Rev (2020) 36:57–64. doi: 10.1016/j.prrv.2020.08.004 PMC7439992 32958428

[41] Freyne B Marchant A Curtis N. BCG-Associated heterologous immunity, a historical perspective: intervention studies in animal models of infectious diseases. Trans R Soc Trop Med Hygiene (2015) 109 (4): 287. doi: 10.1093/trstmh/trv021 25770252

[42] Kato G Kondo H Aoki T Hirono I. Mycobacterium bovis BCG vaccine induces non-specific immune responses in Japanese flounder against nocardia seriolae. Fish Shellfish Immunol (2012) 33:243–50. doi: 10.1016/j.fsi.2012.05.002 22609413

[43] Klitgaard K Friis C Jensen TK Angen Ø Boye M. Transcriptional portrait of actinobacillus pleuropneumoniae during acute disease - potential strategies for survival and persistence in the host. PloS One (2012) 7:e35549. doi: 10.1371/journal.pone.0035549 22530048 PMC3328466

[44] Jensen KJ Hansen MS Heegaard PMH Benn CS Jungersen G. The effect of inactivated mycobacterium paratuberculosis vaccine on the response to a heterologous bacterial challenge in pigs. Front Immunol (2019) 10:1557. doi: 10.3389/fimmu.2019.01557 31333678 PMC6624675

[45] Kaufmann E Khan N Tran KA Ulndreaj A Pernet E Fontes G. BCG Vaccination provides protection against IAV but not SARS-CoV-2. Cell Rep (2022) 38:110502. doi: 10.1016/j.celrep.2022.110502 35235831 PMC8858710

[46] Arts RJW Moorlag SJCF Novakovic B Li Y Wang SY Oosting M. BCG Vaccination protects against experimental viral infection in humans through the induction of cytokines associated with trained immunity. Cell Host Microbe (2018) 23:89–100. doi: 10.1016/j.chom.2017.12.010 29324233

[47] Walk J de Bree LCJ Graumans W Stoter R van Gemert GJ van de V. Outcomes of controlled human malaria infection after BCG vaccination. Nat Commun (2019) 10:874. doi: 10.1038/s41467-019-08659-3 30787276 PMC6382772

