# Supplementary figures and images for: Immunogenicity of Bacillus Calmette-Guérin in pigs: potential as a translational model of non-specific effects of BCG
Source: Front Immunol. 2023 Jul 13;14:1219006. doi: 10.3389/fimmu.2023.1219006 (PMC10374211; doi:10.3389/fimmu.2023.1219006)

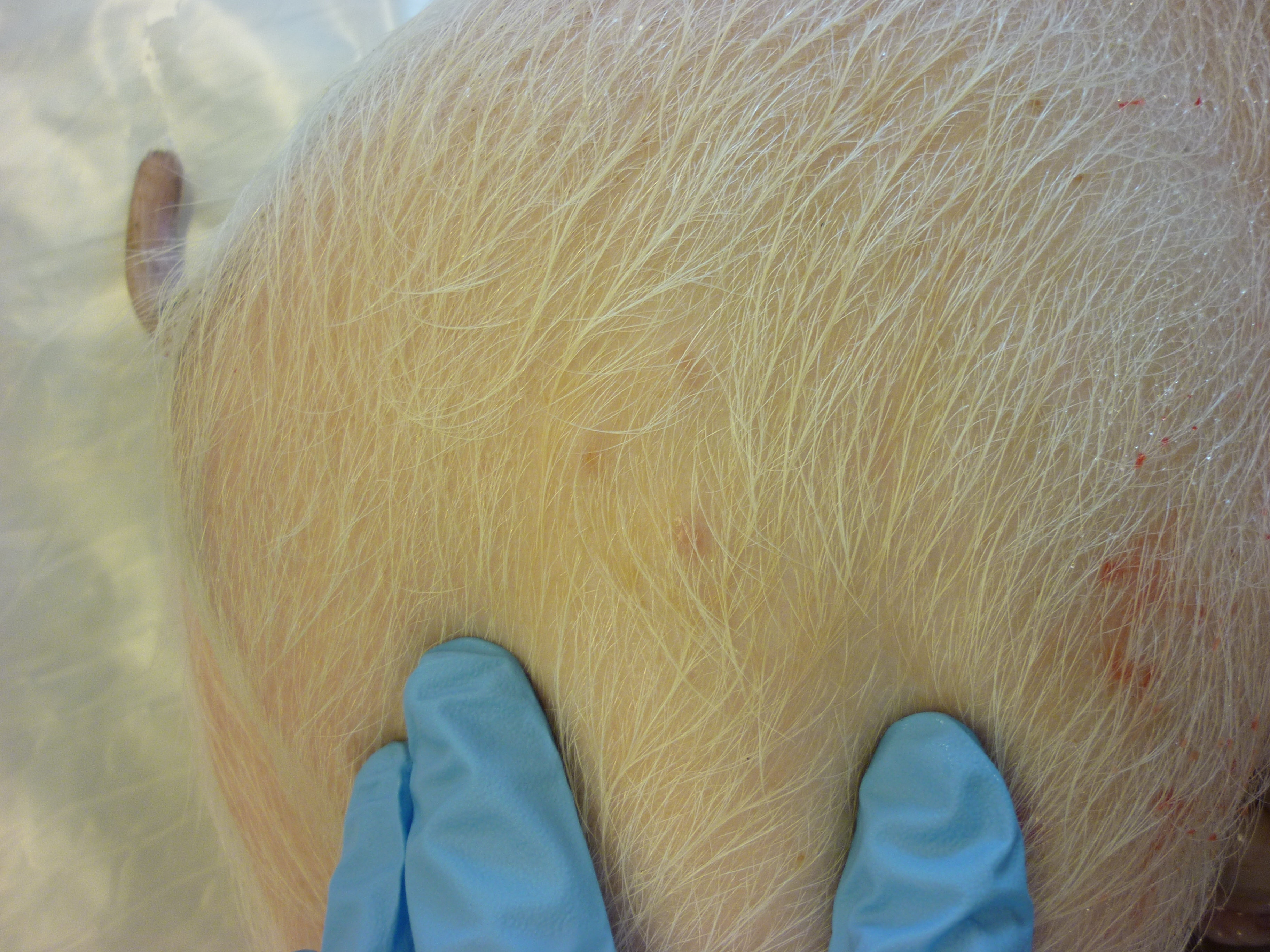

Supplement: Supplementary Figure 1 — Photo of local reaction to high-dose BCG in Experiment C, day 24 (Pig #11). [file Image_1.jpg]

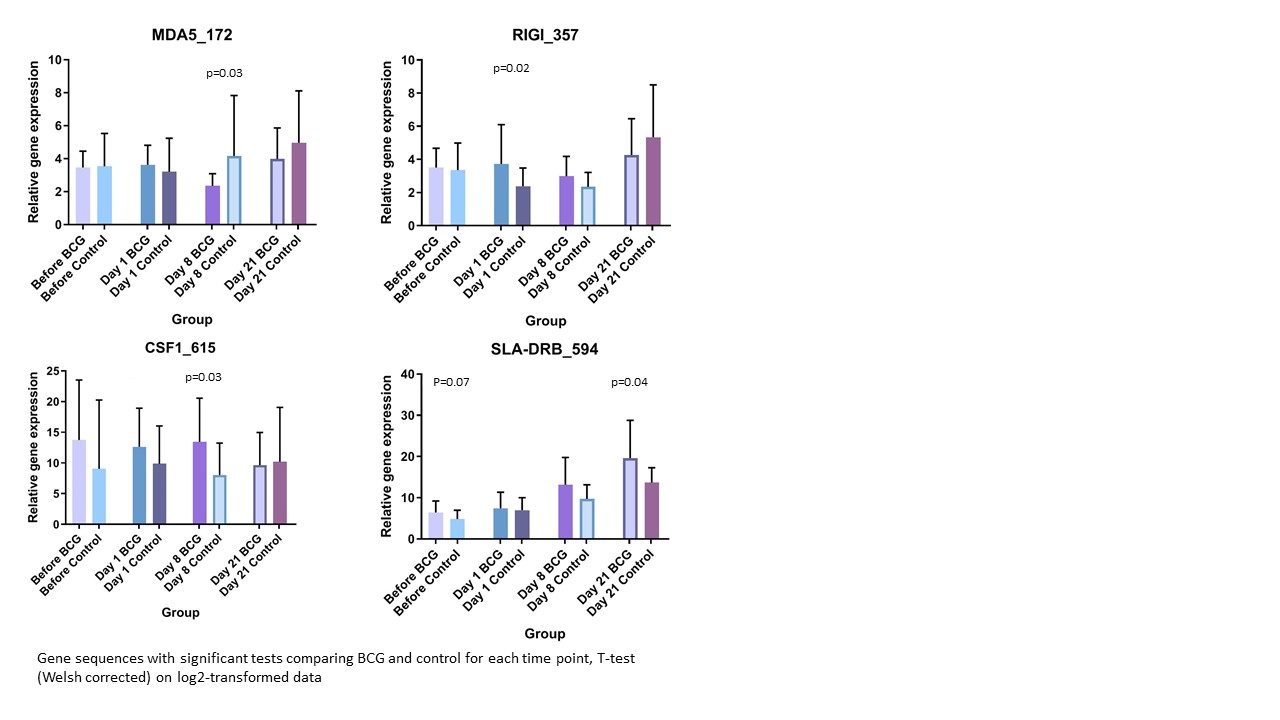

Supplement: Supplementary Figure 2 — Ex vivo gene expression of selected genes in Experiment B. The error bar is the standard deviation. [file Image_2.jpg]

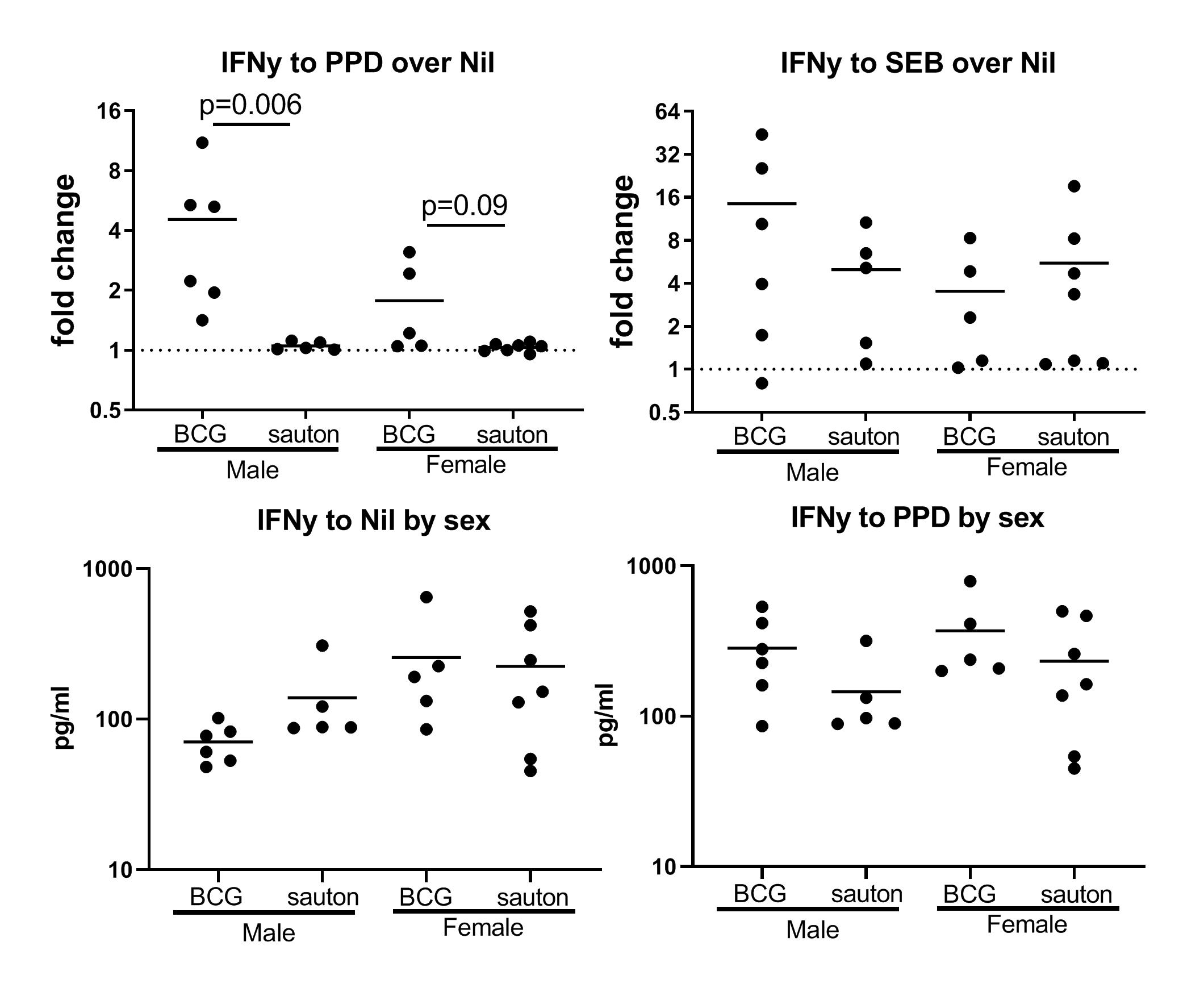

Supplement: Supplementary Figure 3 — In vitro IFN-γ responses, by sex of the animal, in Experiment B. On day 28, undiluted whole blood was stimulated with 10 µg/ml PPDMtb or 1 µg/ml Staphylococcus enterotoxin B (SEB) or unstimulating medium (nil). The value in the two upper panels is relative to the control sample (fold change over unstimulated control). The horizontal bar represents the median. Statistical test using Kruskal-Wallis. BCG male: n=6; Sauton male: n=5; BCG female: n=5; Sauton female: n=7. [file Image_3.jpg]

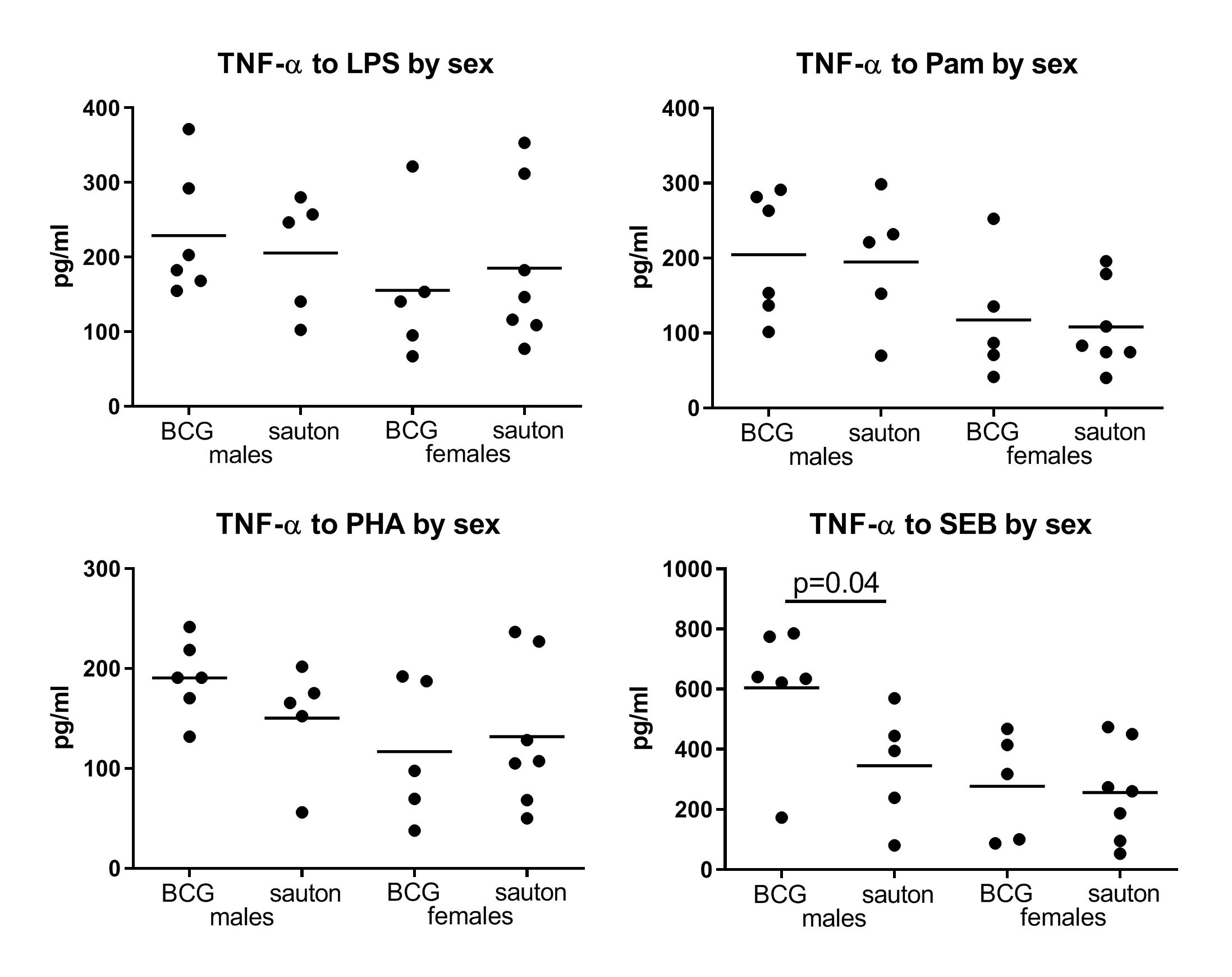

Supplement: Supplementary Figure 4 — In vitro TNF responses, by sex of the animal, in Experiment B. On day 28, undiluted whole blood was stimulated with LPS (10 ng/ml), Pam3CSK4 (1 µg/ml), SEB (1µg/ml) or PHA (2µg/ml) for 22 hours. The horizontal bar represents the median. Statistical test using Kruskal-Wallis. BCG male: n=6; Sauton male: n=5; BCG female: n=5; Sauton female: n=7. [file Image_4.jpg]

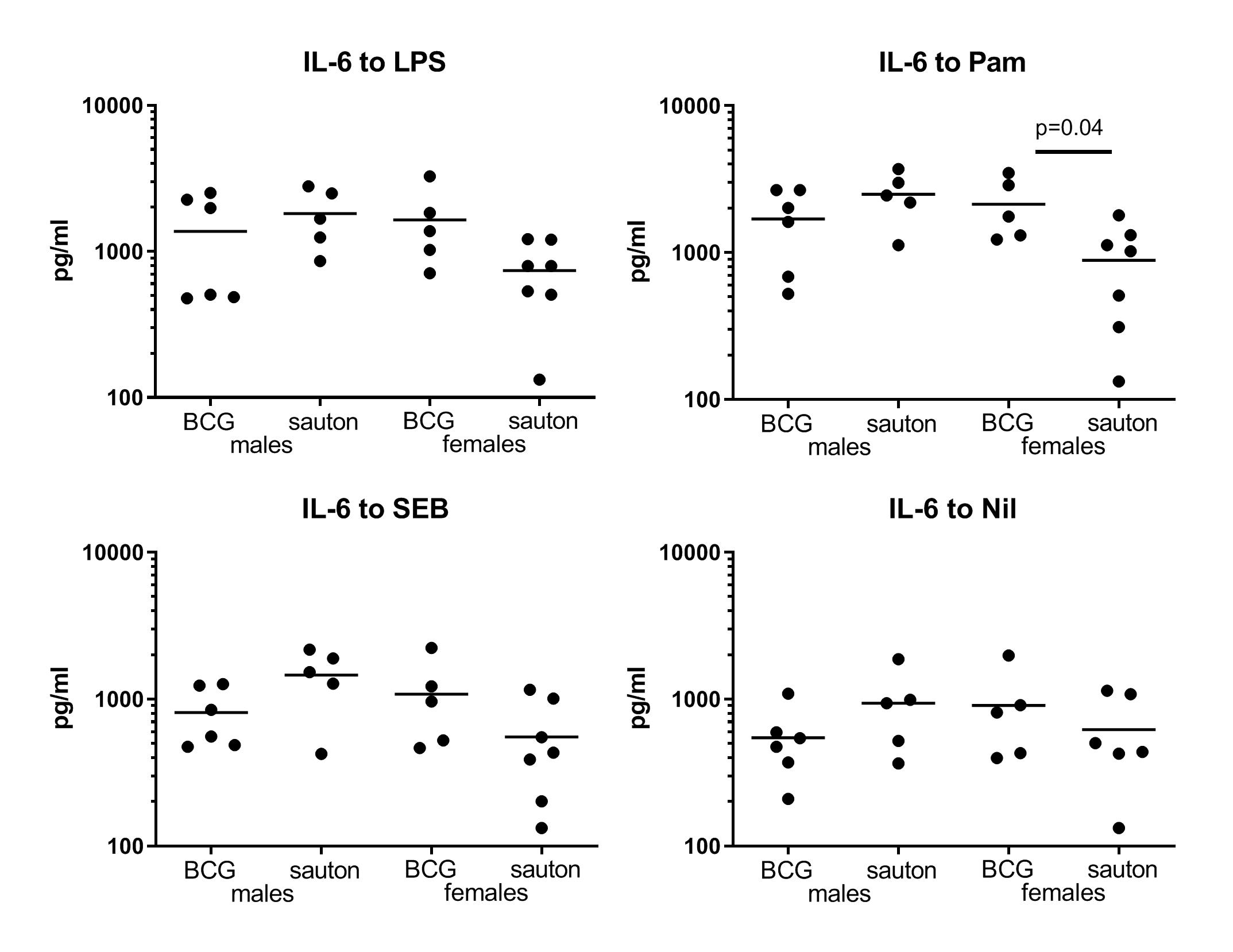

Supplement: Supplementary Figure 5 — In vitro IL-6 responses, by sex of the animal, in Experiment B. On day 28, undiluted whole blood was stimulated with LPS (10 ng/ml), Pam3CSK4 (1 µg/ml), SEB (1µg/ml) or PHA (2µg/ml) for 22 hours. The horizontal bar represents the median. Statistical test using Kruskal-Wallis. BCG male: n=6; Sauton male: n=5; BCG female: n=5; Sauton female: n=7. [file Image_5.jpg]

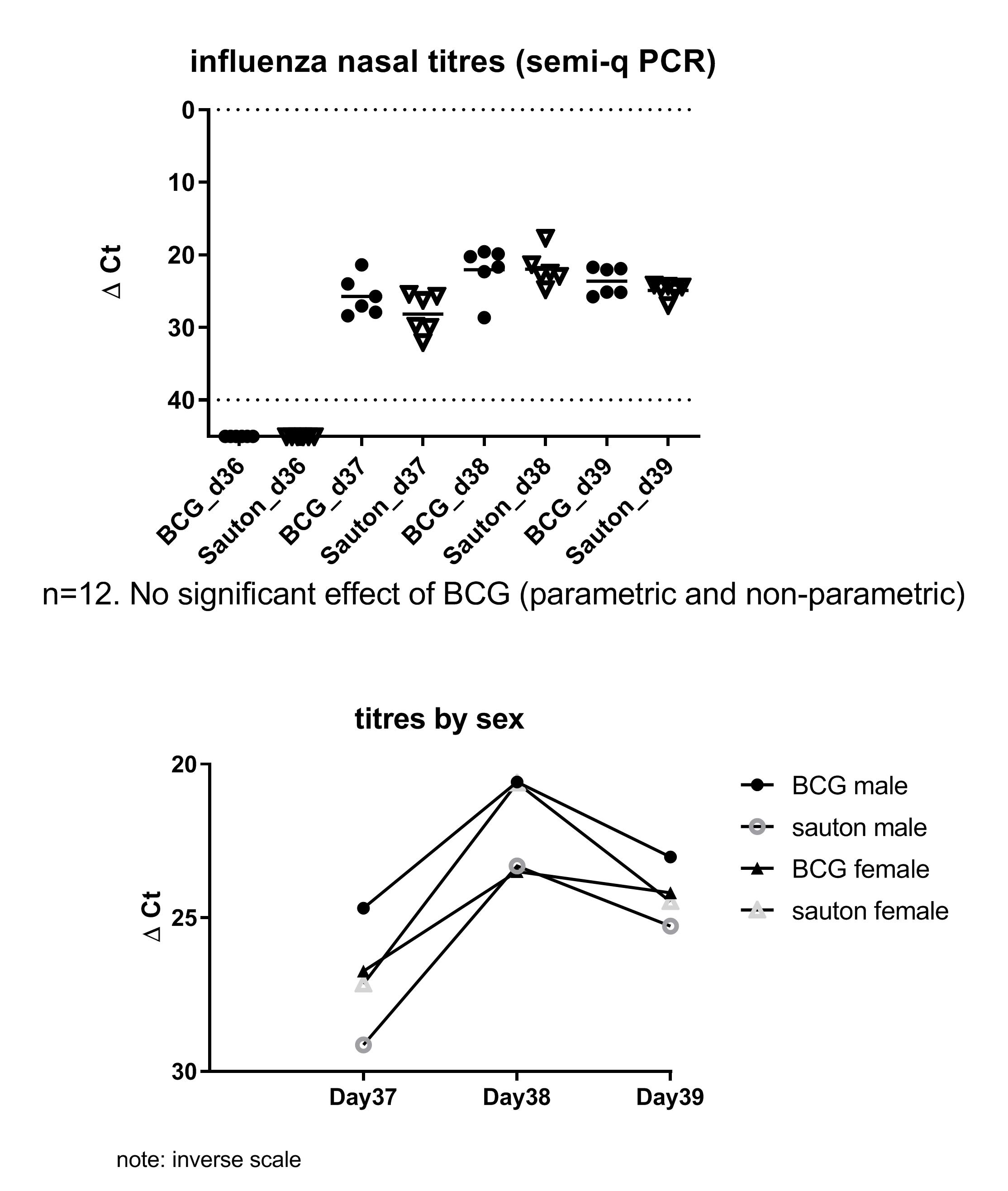

Supplement: Supplementary Figure 6 — Level of influenza virus RNA from nasopharyngeal swabs (Experiment B), quantified using semi-quantitative PCR immediately before (day 36) and after (days 37-39) influenza inoculation. [file Image_6.jpg]
