# Supplementary material for: Immunogenicity of Bacillus Calmette-Guérin in pigs: potential as a translational model of non-specific effects of BCG
Source: Front Immunol. 2023 Jul 13;14:1219006. doi: 10.3389/fimmu.2023.1219006 (PMC10374211; doi:10.3389/fimmu.2023.1219006)
Supplement: Supplementary Table 1 — List of primers for gene expression studies in Experiments B and C. [file Table_1.pdf]

APPENDIX E

List of primers

Table E.1: Table of primers and primer sequences..

Primer name	Info	Sequence
ACTB	actin, beta (ACTB)	F:CTACGTCGCCCTGGACTTC<br>R:GCAGCTCGTAGCTCTTCTCC
B2M	beta-2-microglobulin (B2M)	F:TGAAGCACGTGACTCTCGAT<br>R:CTCTGTGATGCCGGTTAGTG
CD14_8	Cluster of differentiation 14 (CD14)	F:GGGTTCCTGCTCAGATTCTG<br>R:CCCACGACACATTACGGAGT
CD40_9	CD40 molecule, TNF receptor superfamily member 5 (CD40)	F:TGAGAGCCCTGGTGGTTATC<br>R:GCTCCTTGGTCACCTTTCTG
C3_10	complement component 3 (C3)	F:ATCAAATCAGGCTCCGATGA<br>R:GGGCTTCTCTGCATTTGATG
GAPDH	glyceraldehyde-3-phosphate dehydrogenase (GAPDH)	F:ACCCAGAAGACTGTGGATGG<br>R:AAGCAGGGATGATGTTCTGG
HPRTI	hypoxanthine phosphoribosyltransferase 1 (HPRT1)	F:ACACTGGCAAAACAATGCAA<br>R:TGCAACCTTGACCATCTTTG
IL1B_29	interleukin 1 beta (IL1B)	F:CCAAAGAGGGACATGGAGAA<br>R:GGGCTTTTGTTCTGCTTGAG
IL6_35	Interleukin 6 (IL6)	F:TTGCCAGAGAAATCACAGGA<br>R:TGCATGGGACACTGGAAATA
IL8_36	chemokine (C-X-C motif) ligand 8 (interleukin 8) (IL8)	F:TTGCCAGAGAAATCACAGGA<br>R:TGCATGGGACACTGGAAATA
IL8_37	chemokine (C-X-C motif) ligand 8 (interleukin 8) (IL8)	F:GAAGAGAACTGAGAAGCAACAACA<br>R:TTGTGTTGGCATCTTTACTGAGA
IL10_39	interleukin 10 (IL10)	F:TTTAAGGGTTACCTGGGTTGC<br>R:CTCTCTGCCTTCGGCATTAC
IL12_45	interleukin 12 (IL12)	F:GACCAGAAAGAGCCCAAAAAC<br>R:AGGTGAAACGTCCGGAGTAA
IFNA_49	interferon, alpha (IFNA)	F:ATCGTCAGGGCAGAAGTCAT<br>R:CCAGGTGTCTGTCACTCCTTC
IFNG_50	interferon, gamma (IFNG)	F:CCATTCAAAGGAGCATGGAT<br>R:TTCAGTTTCCCAGAGCTACCA
IFNG_51	interferon, gamma (IFNG)	F:GAATTGGAAAGAGGAGAGTGACA<br>R:TCACATCCATGCTCCTTTGA
MYD88_57	myeloid differentiation primary response 88 (MYD88)	F:AGCTGTAGGGGGAATGTGTG<br>R:TCAGCTGGTCTGTGGAGTTG
RPL13A	ribosomal protein L13a (RPL13A)	F:ATTGTGGCCAAGCAGGTACT<br>R:AATTGCCAGAAATGTTGATGC
TLR4_62	toll-like receptor-4 (TLR4)	F:TTTCCACAAAAGTCGGAAGG<br>R:CAACTTCTGCAGGACGATGA
TGFB_70	transforming growth factor beta 1 (TGFB)	F:TCACCGGGGCTGTATTTAAG<br>R:AAGGAAGACCCCAGTCAGGT
TGFB_72	transforming growth factor beta 1 (TGFB)	F:GCAAGGTCCTGGCTCTGTA<br>R:TAGTACACGATGGGCAGTGG
TNF_74	tumor necrosis factor (TNF)	F:CCCCCAGAAGGAAGAGTTTC<br>R:CGGGCTTATCTGAGGTTTGA
IFNB_79	interferon, beta 1, fibroblast (IFNB)	F:AGTTGCCTGGGACTCCTCAA<br>R:CCTCAGGGACCTCAAAGTTCA

Primer name	Info	Sequence
TLR2_100	toll-like receptor 2 (TLR2)	F:GTTTTACGGAAATTGTGAAACTG<br>R:TCCACATTACCGAGGGATTT
IL4_108	interleukin 4 (IL4)	F:GCAAACATGACCTGTTCTGTG<br>R:GCTTCAACACTTTGAGTATTTCTCC
IL27_110	interleukin 27 (IL27)	F:GCCACTTTGCTGAATCACAC<br>R:TGGAGAGGAAGCAGAGTCGT
CXCL10_111	chemokine (C-X-C motif) ligand 10 (CXCL10)	F:CCCACATGTTGAGATCATTGC<br>R:GCTTCTCTCTGTGTTCGAGGA
CCL5_121	chemokine (C-C motif) ligand 5 (CCL5)	F:CTCCATGGCAGCAGTCGT<br>R:AAGGCTTCCTCCATCCTAGC
STAT1_122	signal transducer and activator of transcription 1 (STAT1)	F:CCTTGCAGAATAGAGAACATGATAC<br>R:CCTTTCTCTTGTTGTCAAGCATT
TLR3_123	toll-like receptor 3 (TLR3)	F:ATTGTGCAAAAGATTCAAGGTG<br>R:TCTTCGCAAACAGAGTGCAT
TLR7_124	toll-like receptor 7 (TLR7)	F:AGAAGCCCCTTCAGAAGTCC<br>R:GGTGAGCCTGTGGATTTGTT
TNF_125	tumor necrosis factor (TNF)	F:CACGTTGTAGCCAATGTCAAAG<br>R:GAGGTACAGCCCATCTGTCG
IL10_133	interleukin 10 (IL10)	F:TACAACAGGGGCTTGCTCTT<br>R:GCCAGGAAGATCAGGCAATA
IL1RN_142	interleukin 1 receptor antagonist (IL1RN)	F:TGCCTGTCCTGTGTCAAGTC<br>R:GTCCTGCTCGCTGTTCTTTC
NOD2_145	Nucleotide-Binding Oligomerization Domain Containing 2 (NOD2)	F:GAAAGTCCTGAAGCTGTCCAAC<br>R:CCAGACTTCCAGGATGGTGT
PPIA	peptidylprolyl isomerase A (cyclophilin A) (PPIA)	F:CAAGACTGAGTGGTTGGATGG<br>R:TGTCCACAGTCAGCAATGGT
TBP	TATA-box binding protein (TBP)	F:ACGTTCGGTTTAGGTTGCAG<br>R:CAGGAACGCTCTGGAGTTCT
YWHAE	tyrosine 3-monooxygenase/tryptophan 5-monooxygenase activation protein, epsilon (YWHAE)	F:GCTGCTGGTGATGATAAGAAGG<br>R:AGTTAAGGGCCAGACCCAAT
IL1A_157	interleukin 1 alpha (IL1A)	F:TGTGCTAAATAACCTGGATGAGG<br>R:GGTTCGTCTTCGTTTTGAGC
TLR2_160	toll-like receptor 2 (TLR2)	F:CGGAGGTTGCATATTCCACAG<br>R:TGTGAAAGGGAACAGGGAAC
TLR3_161	toll-like receptor 3 (TLR3)	F:ACATCTACTGAAAGATCCATTGTGC<br>R:TCTTCGCAAACAGAGTGCAT
TLR6_163	toll-like receptor 6 (TLR6)	F:TGGATGTTAGCTCGAATTCTTTG<br>R:GAACCTTGATCCTGGGAGGT
TLR7_164	toll-like receptor 7 (TLR7)	F:GGAAATAGCATCAGCCAAGCTC<br>R:TTCCAGGTTGCGTAGCTCTT
TLR10_165	toll-like receptor 10 (TLR10)	F:TGGAAATCCTGGGTTTGAGTG<br>R:GATGGGCAGGCTACCTTCTT
RIGI_171	DEAD (Asp-Glu-Ala-Asp) box polypeptide 58 (RIGI) (RIGI)	F:ACGAAAGGGGAAGGTTGTCT<br>R:ATGCCTGCAACTTTGTACCC
MDA5_172	Melanoma Differentiation-Associated protein 5 (MDA5)	F:CAGTGTGCTAGCCTGCTCTG<br>R:GCAGTGCCTTGTTTCCTCTC
MYD88_179	myeloid differentiation primary response 88 (MYD88)	F:CCAGACTAAGTTTGCACTCAGC<br>R:AGGATGCTGGGGAACTCTTT
CASP1_184	caspase 1 (CASP1)	F:GAAGGACAAACCCAAGGTGA<br>R:TGGGCTTTCTTAATGGCATC
IRF3_185	interferon regulatory factor 3 (IRF3)	F:GCTACACCCTCTGGTTCTGC<br>R:GAGACACATGGGGACAACCT
MRC1_186	mannose receptor, C type 1 (MRC1)	F:AGAGCACACCTTCCTTTGGA<br>R:CAACACAATCGGCATCTTCA
TLR1_188	toll-like receptor 1 (TLR1)	F:CCTTCAAGACCTTAACACACAGAG<br>R:CAGATTTACTGCGGTGCTGA
IL17_193	interleukin 17A (IL17)	F:TCCAGCAAGAGATCCTGGTC<br>R:AAGAAATATGGCGGACGATG
IL22_194	interleukin 22 (IL22)	F:GAAGTGCTGTTCCCCAACTC<br>R:TACGGCATTGGCTTAGCTTT
IFNA_200	IFNA	F:TTCCAGCTCTTCAGCACAGA<br>R:AGCTGCTGATCCAGTCCAGT

Primer name	Info	Sequence
IL17_210	interleukin 17A (IL17)	F:TACTCCAAACGCTCCACCTC<br>R:AGCATTGATACAGCCCGAGT
IL33_213	interleukin 33 (IL33)	F:AGGCATTCACCAACAAAAGG<br>R:ACAGACCGTTCAAGGTGTCC
TBX21_216	T-box 21 (TBX21)	F:AGGATTCCGGGAGAATTTTG<br>R:GTTGGGGAGGAGAGGAGAGT
IFNB_223	interferon, beta 1, fibroblast (IFNB)	F:AGCACTGGCTGGAATGAAAC<br>R:TCCAGGATTGTCTCCAGGTC
IL6_232	Interleukin 6 (IL6)	F:CCTCTCCGGACAAAACTGAA<br>R:CCTCTCCGGACAAAACTGAA
IL1B_233	interleukin 1 beta (IL1B)	F:TCTCTCACCCCTTCTCCTCA<br>R:GACCCTAGTGTGCCATGGTT
IL18_234	interleukin 18 (IL18)	F:CAATTGCATCAGCTTTGTGG<br>R:TCCAGGTCCTCATCGTTTTC
TLR4_235	toll-like receptor-4 (TLR4)	F:TGGTGTCCCAGCACTTCATA<br>R:CAACTTCTGCAGGACGATGA
IL4_246	interleukin 4 (IL4)	F:TCGGCACATCTACAGACACC<br>R:CTTCTTGGCTTCATGCACAG
CSF2_251	colony stimulating factor 2 (granulocyte-macrophage) (CSF2)	F:CCGAGGAAACTTCCTGTGAA<br>R:GCAGTCAAAGGGGATGGTAA
IL1RAP_276	interleukin 1 receptor accessory protein (IL1RAP)	F:GCATCACCTCCCCAAATCTA<br>R:GTAGCTCCTCTCCCGGTTCT
IRF1_279	interferon regulatory factor 1 (IRF1)	F:TGAAGCTGCAACAGATGAGG<br>R:CTTCCCATCCACGTTTGTCT
IRF2_287	interferon regulatory factor 2 (IRF2)	F:AGCTAGACATGGGTGGGATG<br>R:TACTCCCGGTTGATGCTTTC
IL15_292	interleukin 15 (IL15)	F:CGTCATTTTGCAAGAGTCCA<br>R:TGGACGATAAACTGCTGTTTGC
IFNL_298	Interferon lambda 3 (IFNL)	F:CCTGGAAGCCTCTGTCATGT<br>R:TCTCCACTGGCGACACATT
IDO1_308	indoleamine 2,3-dioxygenase 1 (IDO1)	F:GGGCCCATGACTTACAAGAA<br>R:TTTCCACCAATAGCGAAACC
RIGI_357	DEAD (Asp-Glu-Ala-Asp) box polypeptide 58 (RIGI) (RIGI)	F:TTGCTCAGTGCAATCTGGTC<br>R:CTTCCTCTGCCTCTGGTTTG
OASL_362	2′-5′-oligoadenylate synthetase-like (OASL)	F:TGCGACTGGTAAAACACTGG<br>R:CCCAGGCATAGATGGTCAGT
CSF3_463	colony stimulating factor 3 (CSF3)	F:GCTGCAGATGGAAGACCTG<br>R:GAAGGCCGAGGTGAAGGT
CSF3_464	colony stimulating factor 3 (CSF3)	F:GCTGCAGGAGAGGCTGTG<br>R:GGGGAGGCCCAGAGAGT
MX1_501	MX dynamin-like GTPase 1 (MX1)	F:GCCGAGATCTTTCAGCACCT<br>R:CGGAGGATGAAGAACTGGATGA
CD86_560	CD86 molecule (CD86)	F:CATCGTCTGTGTCCTGCAAC<br>R:CACAGGTGGCTTTGCATCTA
CD86_561	CD86 molecule (CD86)	F:GAACAGGAAGGCGAGTGAAC<br>R:ATCACACTGGGCATCATCAG
ITGAM_576	integrin alpha M (ITGAM)	F:CTCCCCATCAGTGTGGTCTT<br>R:GGAAAGGTTCTGGGAGAAGG
ITGAM_577	integrin alpha M (ITGAM)	F:TAACCCTCTCCCACTCATCG<br>R:AAGCCGAGCTTGTACAGTCC
FCGR3B_587	Fc fragment of IgG, low affinity IIIb, receptor (CD16b) (FCGR3B)	F:CCGAAGTCTGTGGTGATCCT<br>R:CCTGGCACTTCAGAGTCACA
FCGR3B_588	Fc fragment of IgG, low affinity IIIb, receptor (CD16b) (FCGR3B)	F:ACCACATTCCAAATGCAACA<br>R:ACTTTCACAGCCTCCGAAGA
SLA-DRB_593	swine leukocyte antigen DR beta 1 (SLA-DRB)	F:TGACGGTGTATCCTGCAAAG<br>R:GTAGAACCCGGTCACAGAGC
SLA-DRB_594	swine leukocyte antigen DR beta 1 (SLA-DRB)	F:GAGGTCTACAGCTGCCGAG<br>R:ATCTTGCCCTGAGCAGATTC
NOD2_609	Nucleotide-Binding Oligomerization Domain Containing 2 (NOD2)	F:AGCTCGTGGAACATGCTCTT<br>R:CGTCGGTCAATTTGTTGTTG
CSF2_610	colony stimulating factor 2 (granulocyte-macrophage) (CSF2)	F:GCAGCCTCACTAGGCTCAAG<br>R:CAGGAAGTTTCCTCGGTGAG

Primer name	Info	Sequence
CCL5_611	chemokine (C-C motif) ligand 5 (CCL5)	F:ACCACACCCTGCTGTTTTTC<br>R:GAGCACTTGCTGCTGGTGTA
NFKB_612	Nuclear Factor Of Kappa Light Polypeptide Gene Enhancer In B-Cells (2) (NFKB)	F:CCCTGTGAAGACCACCTCTC<br>R:ATCCCGGAGCTCGTCTATTT
NFKB_613	Nuclear Factor Of Kappa Light Polypeptide Gene Enhancer In B-Cells (2) (NFKB)	F:TCCACAAGGCAGCAAATAGA<br>R:AAGCTGAGTTTGCGAAAGGA
CD14_614	Cluster of differentiation 14 (CD14)	F:AAGCTCACCGTGCTTGATCT<br>R:CCTTCCAGGGTCAGGTCAT
CSF1_615	colony stimulating factor 1 (macrophage) (CSF1)	F:CTTCGAGAGCCTTGGACAGT<br>R:ACTGGCAATTCCACCTGTCT
CSF1_616	colony stimulating factor 1 (macrophage) (CSF1)	F:TGCTTCACCAAGGACTACGA<br>R:ACTGGAGGGGCGTCTCATA
TBX21_617	T-box 21 (TBX21)	F:CCCTTCCTTGTGGACTGAGA<br>R:ACACGCCTTCGCTTAGAGTC
ADA_618	adenosine deaminase (ADA)	F:GACATGGGCTTTACCGAAGA<br>R:ATCTGGGAGGAAGCTGGACT
ADA_619	adenosine deaminase (ADA)	F:ACGAGGCCCTTTACACCAG<br>R:GTGAGGTAGCTGGACCAAGG
KMT2A_620	lysine (K)-specific methyltransferase 2A (KMT2A)	F:CCAATGATGAGGAGGAGGAA<br>R:GGCATTGGCAGATCCATACT
KMT2A_621	lysine (K)-specific methyltransferase 2A (KMT2A)	F:CATTGTCATCTTTGCCATGC<br>R:TGCAGGGTAACTTGTTGCTG

Table E.1: Table of primers and primer sequences..
