# Supplementary material for: Immunogenicity of Bacillus Calmette-Guérin in pigs: potential as a translational model of non-specific effects of BCG
Source: Front Immunol. 2023 Jul 13;14:1219006. doi: 10.3389/fimmu.2023.1219006 (PMC10374211; doi:10.3389/fimmu.2023.1219006)
Supplement: Supplementary Table 2 — Weight and temperature in Experiment B. [file Table_2.docx]

Supplementary Table 2. Weight and rectal temperature following randomization to BCG or Sauton (Experiment B)

|  |  | **BCG** | | **Sauton** | |
| --- | --- | --- | --- | --- | --- |
|  | **Day** | **n** | **mean (sd)** | **n** | **mean (sd)** |
| **Weight, kg** | 1 | 16 | 1.6 (0.4) | 14 | 1.9 (0.7) |
|  | 16 | 13 | 4.9 (1.7) | 12 | 6.5 (2) |
|  | 28 | 12 | 8.9 (3.3) | 12 | 11.2 (3) |
|  | 36 | 12 | 10.1 (3.1) | 12 | 12.1 (2.9) |
|  | Ap/Flu, n | 6/6 |  | 6/6 |  |
|  | 37 | 12 | 10.2 (3.3) | 12 | 12.0 (3) |
|  | 38 | 12 | 10.5 (3.4) | 12 | 12.5 (3) |
|  | 39 | 6 | 10.3 (3.2) | 6 | 12.9 (4.4) |
| **Temp, °C** | 36 | 12 | 39.2 (0.3) | 12 | 39.1 (0.4) |
|  | 37 | 12 | 38.7 (0.7) | 12 | 38.6 (0.6) |
|  | 38 | 12 | 38.8 (0.8) | 12 | 38.9 (0.5) |
|  | 39 | 12 | 39.2 (0.3) | 12 | 39.3 (0.6) |

N=32 piglets were randomized to BCG (n=16) or Sauton (n=16) on day 0 of the study. Mean of observations with standard deviation (sd).
